# Tunneling nanotubes induced by pseudorabies virus facilitate viral transmission in neuronal cells to evade the immune system

**DOI:** 10.1186/s12964-026-02765-8

**Published:** 2026-02-28

**Authors:** Yan Kuang, Siqi Fan, Huihui Guo, Haojie Wang, Yafei Li, Hao Zhang, Shaomeng Liu, Dang Wang, Huanchun Chen, Qingyun Liu, Xiangru Wang

**Affiliations:** 1 National Key Laboratory of Agricultural Microbiology, College of Veterinary Medicine, Huazhong Agricultural University, Wuhan, 430070 China; 2 Key Laboratory of Preventive Veterinary Medicine in Hubei Province, The Cooperative Innovation Center for Sustainable Pig Production, Huazhong Agricultural University, Wuhan, 430070 China; 3 Engineering Research Center of Animal Biopharmaceuticals, The Ministry of Education of the People’s Republic of China (MOE), Wuhan, 430070 China; 4 Frontiers Science Center for Animal Breeding and Sustainable Production, Huazhong Agricultural University, Wuhan, 430070 China; 5 Wuhan Keqian Biology Co., Ltd, Wuhan, 430070 China

**Keywords:** Pseudorabies virus, Tunneling nanotubes, Intercellular viral transmission, Immune evasion, Neuropathogenic mechanism

## Abstract

**Background:**

Pseudorabies virus (PRV), an alphaherpesvirus endemic in swine, has recently emerged as a causative agent of fatal human encephalitis, yet its neuropathogenic mechanisms remain largely unexplored. Tunneling nanotubes (TNTs) are actin-rich intercellular membrane extensions that have been found to mediate intercellular communication and facilitate the cell-to-cell transmisssion of viruses.

**Methods:**

Immunofluorescence analysis, confocal imaging, time-lapse photography, in situ sectioning transmission electron microscopy, and scanning electron microscopy were employed to characterize the structure and function of PRV-induced TNTs in SK-N-SH cells widely utilized in neuroscience research. A cell compartment co-culture model was further constructed in this study, and the morphological differences in TNTs, as well as the corresponding viral transmission efficiency under immune pressure, were statistically analyzed and compared between different PRV strains. Additionally, we investigated the role of viral US3 in TNTs formation via proteins overexpression, gene-deleted virus construction and kinase-inactivated US3 protein expression.

**Results:**

We demonstrate that PRV infection induces the extensive formation of TNTs in SK-N-SH cells. These structures act as protected conduits that facilitate efficient cell-to-cell viral transmission, enabling the virus to evade neutralizing antibodies. Critically, a human-isolate variant strain (hSD-1/2019) induced the formation of significantly more, longer and thicker TNTs than a classical PRV strain (Ea). In addition, hSD-1/2019 also exhibited more efficient viral transmission under immune pressure, providing a novel mechanism to explain the enhanced virulence of hSD-1/2019. Further investigation identified that the viral protein US3 and its kinase activity are essential for TNTs formation. However, TNTs induced by viral infection were morphologically distinct from those induced by US3 overexpression alone, suggesting that additional viral factors are required to regulate TNTs maturation and morphology.

**Conclusions:**

Our findings provide novel insights into PRV neuropathogenesis from the perspective of viral transport dynamics, and identify virus-induced intercellular conduits as a potential therapeutic target against infections of PRV and similar neuroinvasive viruses.

**Supplementary Information:**

The online version contains supplementary material available at 10.1186/s12964-026-02765-8.

## Background

Pseudorabies virus (PRV) is a member of the *Alphaherpesvirinae* subfamily, and while it has an extensive host spectrum, swine is its natural host. PRV is widely distributed in pigs worldwide [1]. Even in countries that have eliminated PRV infection from domestic pigs, there remains a risk of the virus spreading from wild animals, particularly wild boars [2–4]. Pigs of all ages are susceptible to PRV infection, resulting in fatal neurological damage and high mortality in newborn piglets, reproductive disorders in breeding pigs, and respiratory diseases in fattening pigs. For decades, PRV infection has caused significant losses in the pig industry in China, and new PRV variant strains that emerged in 2011 have resulted in further outbreaks of swine pseudorabies in China [5]. Compared with the classical strains, the currently circulating PRV variant strains are more virulent in pigs, resulting in more severe clinical symptoms, pathological damage, and higher mortality [6–8].

When non-natural hosts are infected with PRV, fatal encephalomyelitis usually occurs, and the mortality rate is nearly 100% [9]. Historically, PRV was generally considered non-pathogenic to humans; however, PRV was reported in three patients in Europe in 1986 [10]. Recently, 31 clinical cases of PRV infection in humans, defined as human pseudorabies encephalitis, have been reported in China since 2017 [11–22]. The patients all had a history of contact with pigs or pork and showed severe neurological damage and sequelae, and some had died. Given that the PRV strain (hSD-1/2019) isolated from one patient was genetically highly homologous to the variant strains isolated from pigs, it was speculated that the circulating PRV variant strains in pigs could cause human pseudorabies encephalitis [18]. Hence, due to the significant threat that PRV infection poses to both the pig industry and human health, it is essential to elucidate the neuropathogenic mechanism of PRV to develop more effective prevention and control measures.

Tunneling nanotubes (TNTs) are actin-based membrane conduits that connect nearby or distant cells [23, 24], facilitating the direct cell-to-cell transmission of molecules, such as Ca^2+^ [25], proteins [26], and nucleic acids [27], as well as lipid droplets [28] and even organelles [29–31]. Several studies have shown that TNTs can form under pathological conditions [32] and that intercellular communication and molecular transportation mediated by TNTs are essential for the occurrence and development of tumors and neurodegenerative diseases [33–36]. In addition, viral infection has been found to induce TNTs formation, and the viruses could utilize TNTs for cell-to-cell transmission, which protects them from extracellular antibodies, the complement system, and antiviral drugs [37–39]. It has been reported that human immunodeficiency virus (HIV) [40, 41], influenza virus [37, 42], and porcine reproductive and respiratory syndrome virus [38] particles utilize TNTs to infect target cells efficiently. In the case of HIV, virions were found to spread from infected cells to uninfected T cells via TNTs, and TNTs-mediated transmission is reportedly 100–1,000 times more efficient than that via budding [40]. Therefore, TNTs are considered the primary routes of HIV transmission [43], providing a novel target for HIV therapy. Recently, it was found that TNTs also play a crucial role in the transmission of severe acute respiratory syndrome coronavirus 2 (SARS-CoV-2) from epithelial cells to neurons that lack the angiotensin-converting enzyme 2 membrane receptor, a finding that may explain the neural pathogenesis of SARS-CoV-2 [44]. Additionally, there are some reports that several alphaherpesviruses may induce TNTs formation, during which the conserved US3 serine/threonine protein kinase is critical [45, 46]. This cell-to-cell virus transmission through TNTs allows virions to evade the host’s immune system and leads to the reactivation of latent alphaherpesvirus.

Epithelial cells and neurons are the primary target cells of PRV. Previous studies have shown that PRV can induce TNTs formation in animal epithelial cells and fibroblasts [45, 47, 48]. However, it remains unknown whether PRV can induce these structures in neurons, the key cell type involved in the severe neurological disease seen in humans. Moreover, the specific molecular mechanisms driving TNTs formation and the functional role these structures play in PRV neuropathogenesis have not been elucidated. Therefore, this study aimed to determine if PRV induces TNTs in neuronal cells, to identify the viral factors required for this process, and to investigate the role of TNTs in the differential neuropathogenicity of classical and emergent hypervirulent PRV strains.

## Materials and methods

### Cells and viruses

SK-N-SH human neuroblastoma cells (Cat. No. GDC0047; CCTCC, Wuhan, China) were cultured in Minimal Essential Medium (MEM; Gibco, Grand Island, NY, USA) supplemented with 10% fetal bovine serum (FBS; Gibco). SH-SY5Y human neuroblastoma cells (Cat. No. CL-0208; Procell, Wuhan, China) were maintained in a 1:1 mixture of MEM and DMEM F12 medium (Thermo Fisher Scientific, Massachusetts, USA) supplemented with 15% FBS. Rat embryonic neural cells were isolated and cultured from the brain tissues of 15-day-old embryos, as described previously [49]. PK-15 porcine kidney cells (Cat. No. GDC0061; CCTCC) were maintained in Dulbecco’s Modified Eagle Medium (DMEM; Gibco) supplemented with 10% FBS. SK-N-SH cells are treated with retinoic acid (Solarbio, Beijing, China) at a dose of 1µM for 7 days for differentiation. Half of culture medium was replaced every 24 h, with fresh drugs supplemented simultaneously. All cells were maintained in a humidified incubator at 37 °C with 5% CO_2_.

The PRV strain Ea was isolated from an infected pig in China in 1998 [50]. The PRV strain hSD-1/2019 was isolated from the cerebrospinal fluid of a PRV-infected patient in 2019 [18]. The recombinant PRV- monomeric Cherry red fluorescent protein (mCherry) strains (hSD-mCherry and Ea-mCherry) were generated by genetically fusing the mCherry fluorescent protein to the viral capsid protein VP26. The US3-null mutant strains, PRVΔUS3 (hSDΔUS3 and EaΔUS3), were generated by replacing the entire US3 gene with the mCherry coding sequence. All PRV strains used in this study were isolated or constructed and stored in our laboratory.

### Time-lapse imaging

Live-cell time-lapse imaging was performed using either a ZEISS LSM 800 confocal microscope or an EVOS FL Auto inverted fluorescence microscope, both equipped with an environmental chamber maintained at 37 °C and 5% CO_2_. SK-N-SH cells were seeded on glass-bottom culture dishes (Shanghai Longbo Electronic Co., Ltd., Shanghai, China). Once confluent, cell monolayers were infected with PRV-mCherry mutants at a multiplicity of infection (MOI) of 0.1. Following a 2-hour incubation at 37 °C to allow for viral entry, the inoculum was removed and replaced with fresh medium supplemented with a PRV-neutralizing antibody (Wuhan Keqian Biology Co., Ltd., Wuhan, China) to prevent extracellular viral spread. The antibody, with a predetermined neutralizing titer of 1:235, was used at a final dilution of 1:7. Upon the formation of intercellular filaments, live-cell imaging was conducted for 6 h, with images captured at 5 min intervals. The acquired time-lapse sequences were processed using ZEISS ZEN software. Representative videos are provided in the Supplementary Materials (Videos S1–S8).

### Immunofluorescence analysis

For immunofluorescence analysis, cells were fixed by adding 40% formaldehyde solution directly into the culture medium to a final concentration of 4% and incubating for 30 min at room temperature. Following fixation, cells were permeabilized for 20 min with 0.1% Triton X-100 in phosphate-buffered saline (PBS). After three washes with PBS, the cells were incubated with primary antibodies overnight at 4 °C. The following day, cells were washed three times with PBS and incubated with the appropriate secondary antibodies for 1 h at 37 °C. Nuclei were then counterstained with 4’,6-diamidino-2-phenylindole (DAPI; biosharp) for 5 min. Finally, coverslips were mounted using Antifade Mounting Medium (biosharp), and images were acquired using a ZEISS LSM 800 or a Nikon N-STORM confocal microscope.

The following primary and secondary antibodies were used: mouse anti-PRV-glycoprotein B (gB) monoclonal antibody (a gift from Wuhan Keqian Biology Co., Ltd; diluted 1:100), α-tubulin mouse monoclonal antibody (CST, 3873P; diluted 1:2,000), and rabbit anti-mouse IgG/Alexa Fluor 594 antibody (Bioss, bs-0296R-AF594; diluted 1:500). F-actin was stained with Phalloidin-Alexa Fluor 488 (Beyotime, C2201S; diluted 1:200). All antibodies were diluted in PBS containing 5% bovine serum albumin (BSA).

### Measurement of TNT length and width

Brightfield images for TNT length measurement were acquired using an Olympus IX53 fluorescence microscope (Olympus, Tokyo, Japan). TNT lengths were measured from 10 randomly selected fields of view per experiment, across three independent experiments, using cellSens Entry software (Olympus). For TNT width measurement, scanning electron microscopy (SEM, Hitachi SU8010, Tokyo, Japan) was employed to acquired images of TNTs induced by PRV strains in SK-N-SH cells. TNT width were measured from 23 randomly selected fields of view per experiment, across three independent experiments, using ImageJ2 (National Institutes of Health).

### Construction of US3 expression plasmids and kinase-inactivated US3 expression plasmid

The open reading frame (ORF) of the viral US3 gene was amplified by Polymerase Chain Reaction (PCR) using the forward primer 5’-CCCAAGCTTatgctggcgattggagatg-3’ and the reverse primer 5’-CGGGATCCtacggtccacatcatccaaagttgag-3’. The amplified US3 ORF was subsequently cloned into the p3×FLAG-CMV-14 expression vector (Biofeng, E7908). Correct clones were verified by PCR, restriction enzyme digestion, and sequencing. Protein expression was confirmed by Western blotting (Fig S1). The resulting expression plasmids were designated hSD-US3 and Ea-US3. The kinase-inactivated US3 plasmids were construscted by replacing lysine (K) at position 136 of the US3 protein with glycine (G), or substituting aspartic acid (D) at position 221 with alanine (A), as described before [51]. The kinase-inactivated US3 expression plasmid was commercially synthesized by company (TsingkeBiotecnology, Beijing, China). Western blotting validation of US3 protein expression is provided in Fig S1.

### Construction of US3-knockout PRV strains

The homologous recombination donor plasmid was constructed based on the pcDNA3.1(+) vector (Biofeng, 69909). The mCherry gene was amplified using the forward primer 5’-GGGGTACaggcagcaggcgagga-3’ and the reverse primer 5’-GGAATTCttgtacatcatcatctcgtccatccg-3’. The upstream homologous arm (US3-UP) was amplified using primers 5’-CCCAAGCTTatgccgacgccggaatcc-3’ (forward) and 5’-GGGGTACCgtgcatgatgcatcccgtgc-3’ (reverse). The downstream homologous arm (US3-DOWN) was amplified using primers 5’-GGAATTCaacggcccggctccgagc-3’ (forward) and 5’-GCTCTAGAcacgtcgtagtcgtc-3’ (reverse). All PCR amplifications were performed using PrimeSTAR^®^ Max DNA Polymerase (Takara, R045B). Following verification, the US3-UP, mCherry, and US3-DOWN fragments were sequentially ligated into pcDNA3.1(+) to generate the donor plasmid, pcDNA3.1(+)-US3-UP-mCherry-US3-DOWN.

PK-15 cells, seeded in six-well plates and grown to 70–80% confluence, were co-transfected with 8 µg of the donor plasmid and 4 µg of PRV genomic DNA (prepared as previously described [18]) using Lipofectamine^®^ 2000 (Life Technologies, 11668027). The cells were incubated at 37 °C in 5% CO_2_ for 72 h, or until cytopathic effects (CPE) accompanied by red fluorescence were observed. Recombinant viruses were isolated and purified through 3–5 rounds of plaque purification to obtain the hSDΔUS3 and EaΔUS3 strains.

### Transfection and co-transfection

Transient transfections were performed using jetPRIME^®^ (Polyplus, 101000046) according to the manufacturer’s instructions. Briefly, SK-N-SH cells at 80% confluence in a six-well plate were transfected with 2 µg of plasmid. After 4 h of incubation at 37 °C and 5% CO_2_, the transfection medium was replaced with fresh MEM containing 3% FBS, and the cells were incubated for an additional 48 h before subsequent experiments. For co-transfection, 1 µg of hSD-US3 or Ea-US3 plasmid was co-transfected with 1 µg of pEGFP-N1 (Biofeng, 6085-1) using the same protocol. Twenty-four hours post-transfection, tests was performed.

### Cell compartment co-culture model

To analyze virus transmission in the presence of neutralizing antibodies, a cell compartment co-culture model was established. Donor SK-N-SH cells were seeded onto rectangular glass slides (CITOGLAS, 10212424 C) and grown to confluence. These confluent donor cells were then infected with PRV-mCherry strains (MOI = 0.1) and incubated for 12 h. Subsequently, the glass slide with the adherent infected cells was transferred to a circular culture dish containing a monolayer of uninfected SK-N-SH receptor cells. A PRV-neutralizing antibody (1:7 final dilution) was present in the co-culture medium throughout the experiment to inhibit free virus transmission. The co-culture system was observed using an EVOS FL Auto inverted fluorescence microscope, and images were captured at various distances from the donor cells.

### In situ sectioning transmission electron microscopy (TEM)

A confluent monolayer of SK-N-SH cells was seeded in 14 mm Glass Bottom Cell Cuture Dish (Cat.No. J40141, JING’AN Biotechnology, Shanghai, China) and infected with hSD-1/2019 (MOI = 0.1), with or without the addition of neutralizing antibodies with complete neutralizing activity to the culture supernatant. When filamentous structures were observed in the cells, the samples were submitted to the Institutional Center for Shared Technologies and Facilities of Wuhan Institute of Virology for in situ sectioning TEM analysis using a Thermo Fisher Talos L120C (Thermo Fisher Scientific, Massachusetts, USA).

### Statistics

Data are presented as mean ± standard deviation (SD) from at least three independent experiments. Statistical significance was assessed using a two-sided Student’s t-test in GraphPad Prism (version 6.0). A *P*-value < 0.05 was considered statistically significant. Significance levels are denoted in figures as follows: ****, *P* < 0.0001; ns, *P* > 0.05 (not significant).

## Results

### PRV infection induces the formation of TNTs in neuronal cells

**Fig. 1:**
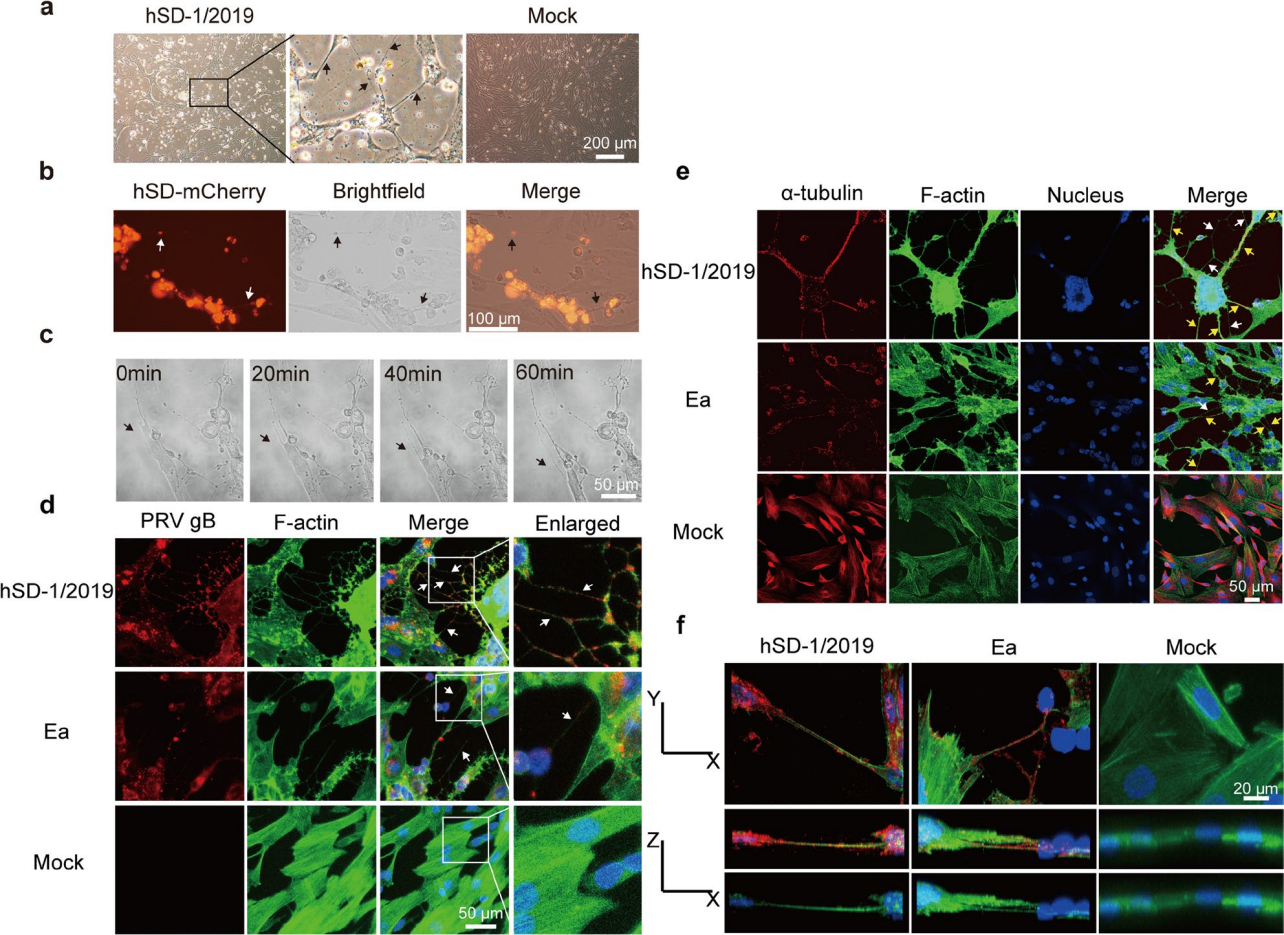
PRV infection induces the formation of TNTs in SK-N-SH cells. **a**. Infection with the PRV hSD-1/2019 strain (MOI = 0.1) induces distinct filamentous structures between SK-N-SH cells. The image in the larger box is a magnified view of the region indicated in the smaller box. **b**. Following infection of SK-N-SH cells with hSD-mCherry virions, red fluorescence was observed within the lumens and vesicular structures of the filaments. **c**. Representative time-lapse microscopy images illustrating the formation process of filamentous structures in SK-N-SH cells infected with the hSD-1/2019 strain (MOI = 0.1) (see also S2 Video). Arrows in all images indicate the filamentous structures. **d**. PRV-induced filamentous structures contain F-actin (green) and PRV gB protein (red); nuclei are counterstained blue. White arrows indicate the filaments. **e**. PRV-induced filaments contain α-tubulin, particularly within longer and thicker structures. α-tubulin: red; F-actin: green; nuclei: blue. White arrows point to filaments containing only F-actin, while yellow arrows indicate filaments containing both F-actin and α-tubulin. **f**. Three-dimensional reconstruction of infected cells reveals that the filaments lack contact with the underlying substrate. PRV gB: red; F-actin: green; nuclei: blue. Images were acquired using a confocal microscope (ZEISS LSM 800 or Nikon N-STORM)

Given that PRV infection in humans invariably presents with severe neurological symptoms, we investigated PRV infection in SK-N-SH, SH-SY5Y, and mouse primary neurons. Infection with the human-isolate PRV strain, hSD-1/2019, induced the formation of numerous, long, filamentous protrusions connecting adjacent cells, which were absent in uninfected controls (Fig. 1a, S1 Video). When cells were infected with a fluorescently labeled virus (hSD-mCherry), the filaments and vesicular structures contained viral particles, with vesicular structures demonstrated very intense fluorescence (Fig. 1b). Time-lapse microscopy confirmed these filaments could extend over 100 μm and remained stable for extended periods (Fig. 1c, S2 Video).

To confirm these structures were authentic tunneling nanotubes (TNTs), we characterized them based on established criteria. TNTs are characterized by four key features: a membranous conduit mediating direct linear connections between cells; the presence of actin filaments and, in certain instances, microtubules; no physical contact with the underlying substrate on which the cells are grown; and the capacity to enable direct intercellular crosstalk via the transfer of molecules or organelles [52–54]. Immunofluorescence analysis revealed that the filaments induced by both the variant hSD-1/2019 and classical Ea strains were positive for F-actin and viral gB protein (Fig. 1d). While all filaments contained F-actin, a subpopulation of thicker, longer filaments also contained α-tubulin, suggesting a more stable cytoskeletal architecture (Fig. 1e) [47]. Crucially, 3D reconstruction of confocal z-stacks confirmed that these intercellular bridges did not make contact with the underlying culture substrate (Fig. 1f). After treating with retinoic acid for 7 days, a subset of SK-N-SH cells exhibited neuronal morphological characteristics (Fig S2a) and expressed the neuronal markers TUBB3 and MAP2 (Fig S2b, S2c). Following infection with hSD-1/2019 strain, these differentiated SK-N-SH cells also formed actin-rich filamentous structures that connect the differentiated neurons (Fig S2d). In addition, PRV infection was also found to induce TNT formation in SH-SY5Y cells (another commonly used cell model in neuroscience research) and mouse primary neurons (Fig S3). Collectively, these findings support that PRV infection induces the formation of TNTs in neuronal cells.

### The viral kinase US3 and its kinase activity are essential for PRV-induced TNTs formation

**Fig. 2:**
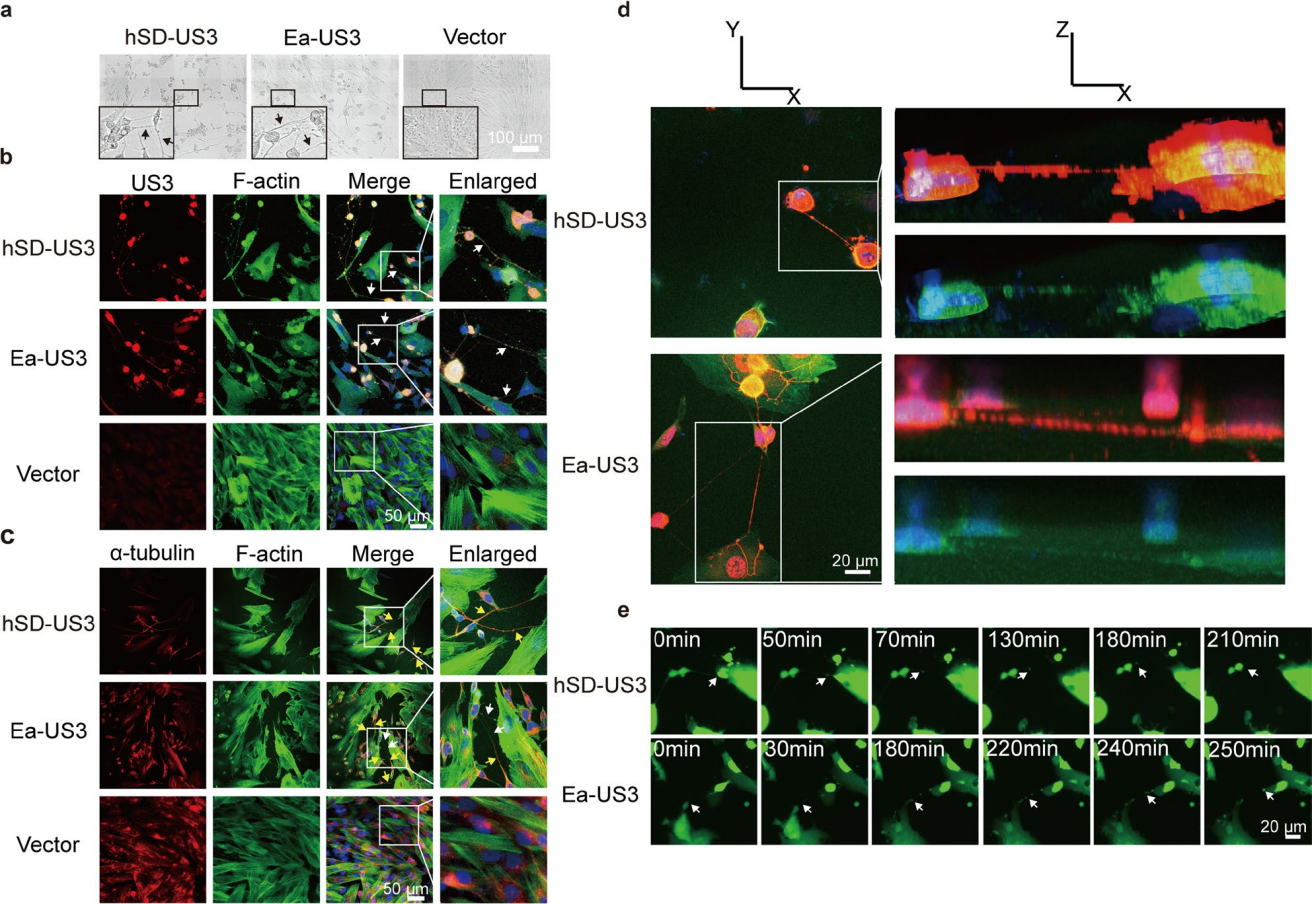
The viral US3 protein induces TNTs formation in SK-N-SH cells. **a**. Overexpression of US3 in SK-N-SH cells induces the formation of numerous filamentous structures between cells. The image in the larger box is a magnified view of the region indicated in the smaller box. Arrows indicate filaments. **b**. Immunofluorescence images of US3-transfected SK-N-SH cells show that the resulting filaments contain F-actin. US3: red; F-actin: green; nuclei: blue. Arrows indicate filaments. **c**. Immunofluorescence images of US3-transfected SK-N-SH cells reveal the presence of α-tubulin within the filaments. α-tubulin: red; F-actin: green; nuclei: blue. White arrows point to filaments containing only F-actin, while yellow arrows indicate filaments containing both F-actin and α-tubulin. **d**. Three-dimensional reconstruction of US3-transfected cells indicates that the induced filaments do not make contact with the underlying substrate. US3: red; F-actin: green; nuclei: blue. **e**. SK-N-SH cells were co-transfected with plasmids expressing viral US3 and EGFP, and subsequently observed by time-lapse microscopy. Representative images show that US3-induced TNTs mediate the transport of EGFP between SK-N-SH cells (see also S4 and S5 Videos). Arrows indicate EGFP. Immunofluorescence images were acquired using a confocal microscope (ZEISS LSM 800 or Nikon N-STORM), and time-lapse microscopy was performed using an inverted fluorescence microscope (EVOS FL Auto)

Previous studies have implicated the conserved alphaherpesvirus kinase US3 in remodeling the host cytoskeleton in epithelial cells and fibroblasts [47, 48]. We therefore investigated its role in PRV-induced TNTs formation in SK-N-SH cells. First, to determine if US3 is sufficient to induce these structures, we transfected SK-N-SH cells with plasmids expressing either hSD-US3 or Ea-US3. Compared to vector-only control cells, which showed no such structures, cells overexpressing either US3 protein generated numerous intercellular filaments (Fig. 2a, S3 Video). To characterize these filaments, we performed immunofluorescence staining. US3 protein was observed to localize within the cytoplasm, nucleus, and the filaments themselves (Fig. 2b). These US3-induced filaments were positive for F-actin, and the majority also stained positive for α-tubulin, indicating a cytoskeletal composition similar to virus-induced TNTs (Fig. 2b and c). We then co-transfected cells with a US3 plasmid and a plasmid expressing soluble Enhanced Green Fluorescent Protein (EGFP). Three-dimensional reconstruction confirmed that these filaments did not adhere to the underlying substrate, and time-lapse imaging captured the transfer of EGFP from a donor to a recipient cell through these connections, confirming they are functional conduits (Fig. 2d and e, S4 and S5 Videos). These data demonstrate that expression of the US3 protein alone is sufficient to induce the formation of bona fide TNTs in SK-N-SH cells.

**Fig. 3:**
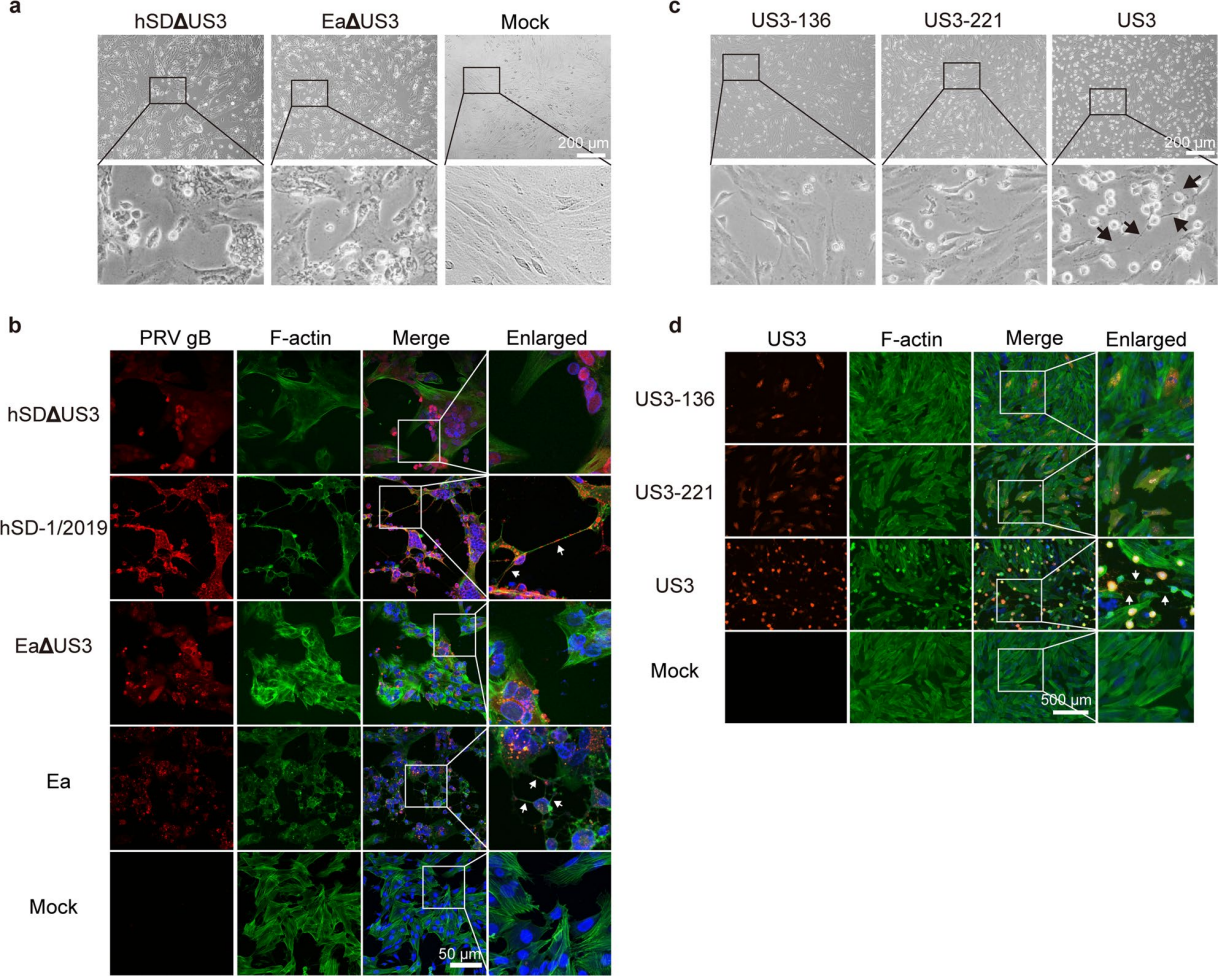
US3-knockout PRV and kinase-inactivated US3 protein fail to induce TNTs formation in SK-N-SH cells. US3-null mutants, denoted as PRVΔUS3 (hSDΔUS3 and EaΔUS3), were constructed by replacing the US3 gene with mCherry. **a** Brightfield images and (**b**) immunofluorescence images of SK-N-SH cells infected with the wildtype strains (hSD-1/2019 and Ea) showed widespread TNTs network, while the hSDΔUS3 or EaΔUS3 strain showed no formation of TNTs between cells. Immunofluorescence images were acquired using a Nikon N-STORM microscope. **c**. Brightfield images and **d**. immunofluorescence images of SK-N-SH cells transfected with the wildtype US3 plamid showed abundant filaments, while those transfected with kinase-inactivated US3 plasmids showed no filaments

Next, to determine if US3 is necessary for this process during infection, we generated US3-knockout mutants for both the hSD-1/2019 and Ea strains by replacing the US3 gene with an mCherry reporter (hSDΔUS3 and EaΔUS3). SK-N-SH cells were infected with these mutant viruses and monitored for filament formation. In stark contrast to the widespread TNTs network observed during wild-type infection, cells infected with either the hSDΔUS3 or EaΔUS3 mutant completely failed to produce any TNT-like structures, despite showing clear signs of infection as indicated by mCherry expression (Fig. 3a and b). Moreover, in sharp contrast to the results of transfection with the US3 eukaryotic expression plasmid (Fig. 2), transfection of SK-N-SH cells with the kinase-inactivated US3 plasmid failed to induce TNT formation (Fig. 3c and d).

Collectively, these gain-of-function (overexpression) and loss-of-function (US3 knockout or kinase inactivate) experiments unequivocally establish that the viral kinase US3 is both necessary and sufficient for the initiation of TNTs formation in PRV-infected SK-N-SH cells.

### TNTs mediate viral transmission and confer protection from neutralizing antibodies

**Fig. 4:**
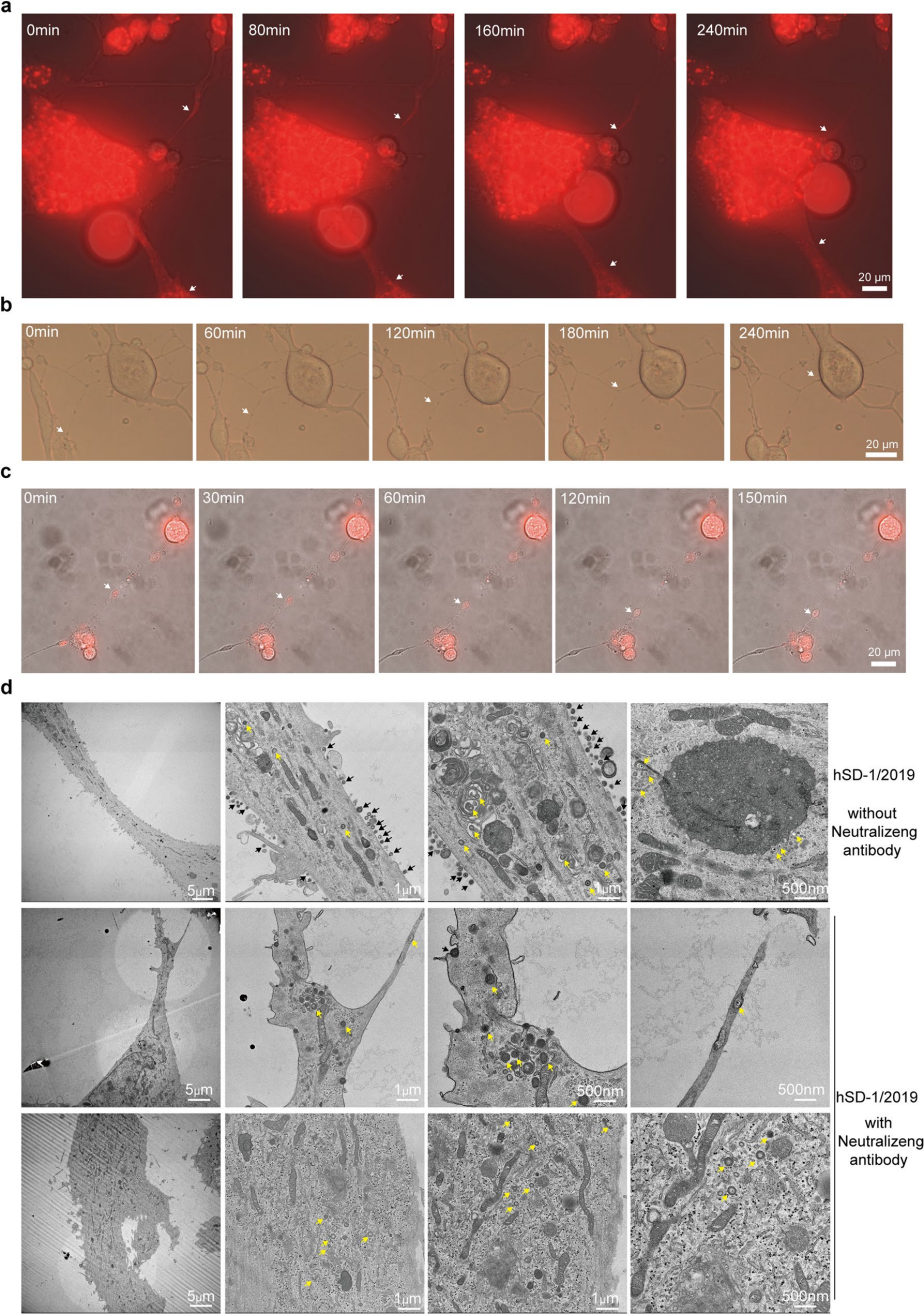
PRV utilizes TNTs for cell-to-cell transmission. SK-N-SH cells were infected with mCherry-labeled PRV virions (MOI = 0.1) for 24 h. Subsequently, time-lapse microscopy was performed using either a confocal microscope (ZEISS LSM 800) or an inverted fluorescence microscope (EVOS FL Auto). **a**. Representative images showing the transmission of mCherry-labeled virions between cells through TNTs (see also S6 Video). **b**. Representative images showing the rapid transport of multiple vesicular structures within TNTs between cells (see also S7 Video). **c**. Representative images illustrating the transmission of mCherry-labeled virions via the movement of vesicular structures within TNTs (see also S8 Video). **d**. In situ sectioning transmission electron microscopy illustrated TNTs structures. Arrows indicate the transmitted mCherry-labeled virions or the transported vesicular structures

We next investigated the functional role of these TNTs in viral dissemination. Time-lapse imaging of cells infected with hSD-mCherry revealed the rapid, directional transport of fluorescent viral puncta through the nanotube interior of TNTs, connecting distant infected cells (Fig. 4a, S6 Video). In some cases, virions appeared to be transported within vesicular “gondolas”, which were mostly observed in the thinner TNTs (Fig. 4b and c, S7 and S8 Videos). In situ sectioning TEM revealed the presence of abundant structures, including mitochondria, endoplasmic reticulum, and viral particles, within the lumen of TNTs (Fig. 4d). By TEM, when there was no neutralizing antibody in the culture supernatant, viral particles were observed both inside and outside the TNTs; however, after the addition of neutralizing antibody, the viral particles outside the TNTs disappeared, and viral particles could only be detected inside the TNT lumen.

**Fig. 5:**
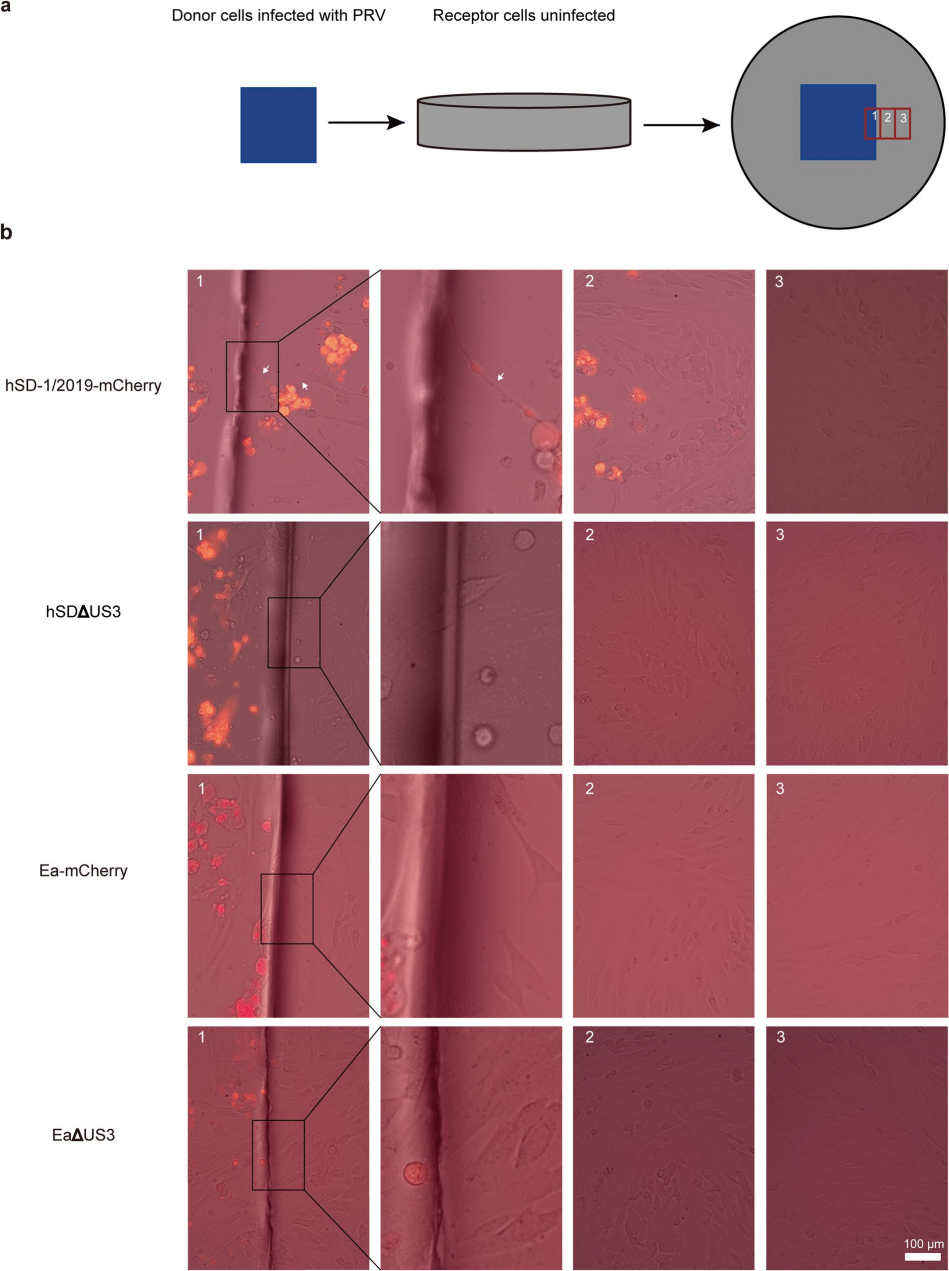
PRV-induced TNTs enhance long-distance viral transmission in SK-N-SH cells in the presence of neutralizing antibody. **a** Schematic diagram of the cell compartment co-culture model. Confluent donor SK-N-SH cells on a glass slide were infected with mCherry-labeled PRV strains at an MOI of 0.1 for 12 h. The glass slide was then transferred to a culture dish containing confluent, uninfected recipient SK-N-SH cells. Excess neutralizing antibody was added during co-culture to inactivate virions released via budding. The culture medium was replaced with fresh medium containing neutralizing antibody every 12 h. Images were captured at various distances from the donor cells to assess viral transmission efficiency. **b **Cell-to-cell transmission of PRV-mCherry and PRVΔUS3 virions between donor and recipient cells in the co-culture model. Images labeled 1–3 were captured at three different distances from the donor cells. The efficiency of viral transmission was determined based on fluorescence intensity and transmission distance in recipient cells

To further test whether this mode of transfer allows for immune evasion, we developed a cell compartment co-culture model where infected donor cells are separated from a monolayer of uninfected recipient cells, as decribed in Materials and methods. A schematic of the model is shown in Fig. 5a. The entire system was cultured in the presence of a high concentration of PRV-neutralizing antibodies to block all extracellular virus-mediated spread. Under these conditions, only the wild-type hSD-mCherry strain, which form abundant TNTs, were able to transmit to recipient cells (Fig. 5b). In stark contrast, the hSDΔUS3 and EaΔUS3 strains, which cannot form TNTs, as well as the wild type Ea-mCherry, failed to establish infection in the recipient cell layer (Fig. 5b). This demonstrates that the PRV strain that induces abundant TNTs shows efficient cell-to-cell viral spread, shielding the virus from the immune pressure of neutralizing antibodies. Moreover, hSD-mCherry mediated a significantly more efficient viral spread under immune pressure than Ea-mCherry, which is consistent with the morphological difference of TNTs induced by the two strains described below.

### The variant hSD-1/2019 strain induces a morphologically superior TNTs network than the classical Ea strain

**Fig. 6:**
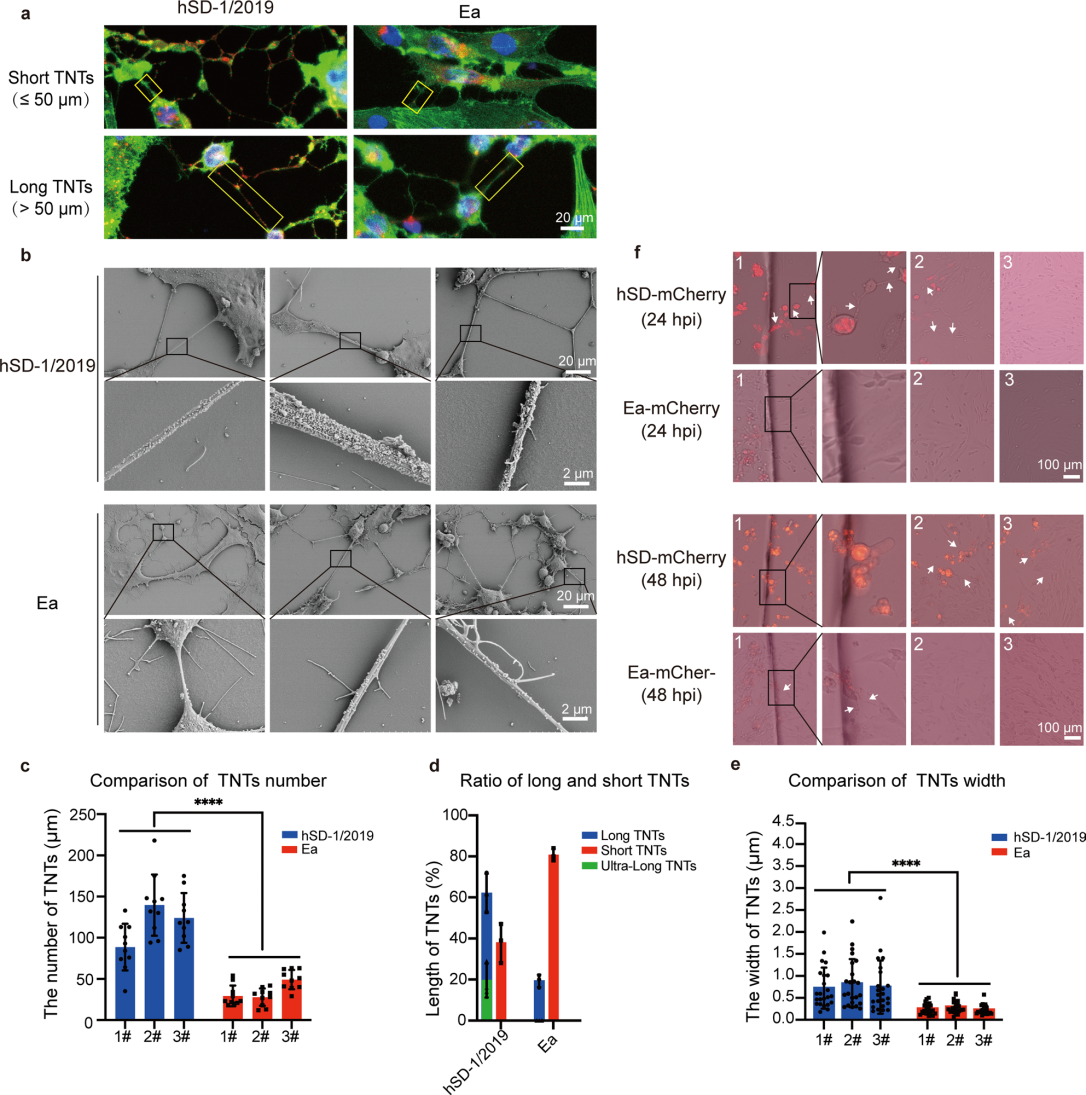
The PRV variants induce more/longer TNTs and more efficient viral transmission under immune pressure. **a** For quantification of TNT length, TNTs were classified as either short (≤ 50 μm) or long (> 50 μm). Cells infected with either the hSD-1/2019 or Ea strain exhibited both short and long TNTs. **b** Brightfield images showing the different morphologies of TNTs induced by infection with the hSD-1/2019 and Ea strains. The image in the larger box is a magnified view of the region in the smaller box. **c**, **d** and **e**. Comparative analysis of the number (**c**), length (**d**) and width (**e**) of TNTs detected in cells infected with the hSD-1/2019 and Ea strains. TNTs were manually counted in three separate experiments, with 10 fields of view randomly selected per experiment. The number of TNTs is presented as the average count from the 10 fields, and each dot represents a field (**c**). TNT length is presented as the average ratio of short to long TNTs from the three replicate experiments, and each dot represents one experiment (**d**). **e** TNTs were manually counted in three separate experiments, with 23 fields of view randomly selected per experiment. The width of TNTs is presented as the average count from the 23 TNTs. **f** In the cell compartment co-culture model, the cell-to-cell transmission efficiencies of the hSD-1/2019 and Ea strains were observed and compared at 24 h and 48 h post co-culture.

We consistently observed that the TNTs networks formed by the variant and classical strains appeared morphologically different. To rigorously test this, we quantified the key structural parameters, including the number, length and width of TNTs in infected SK-N-SH cells. TNTs were defined as short (≤ 50 μm) or long (> 50 μm), which could be observed in SK-N-SH cells infected with hSD-1/2019 and Ea strains (Fig. 6a). TNT width was measured by scanning electron microscopy (Fig. 6b). The parameters were quantified in three replicate experiments, with 10 (length measurement) or 23 (width measurement) observation fields randomly selected in each experiment (Fig. 6c, d and e). Infection with the hSD-1/2019 variant induced a significantly more robust network, producing over three times more TNTs per field of view than the classical Ea strain (118 vs. 35, respectively) (Fig. 6c). The hSD-1/2019 TNTs were also substantially longer and more complex: 62% of them exceeded 50 μm in length, with a third of these classified as “ultra-long” (> 100 μm)—a phenotype rarely observed with Ea infection (Fig. 6d). Additionally, the TNTs induced by hSD-1/2019 infection were visually thicker than those induced by Ea infection (Fig. 6b). The measurement data showed that the average width of TNTs induced by hSD-1/2019 was 0.76 μm, which was significantly thicker than that of TNTs induced by strain Ea (0.26 μm) (Fig. 6e).

When tested in the co-culture model with neutralizing antibodies, the hSD-1/2019 strain demonstrated potent long-distance transmission, with viral fluorescence appearing in multiple distal regions of the recipient cell layer by 48 h. In contrast, transmission by the Ea strain was inefficient and remained confined to cells in the immediate vicinity of the donor slide (Fig. 6f). Overall, the variant strain hSD-1/2019 builds a more extensive and robust TNTs network and shows more efficient and distant viral spread under immune pressure. The association between this superior network architecture and the enhanced functional capacity merits further in-depth investigation.

### The enhanced TNTs phenotype of hSD-1/2019 is orchestrated by additional viral factors beyond US3

**Fig. 7:**
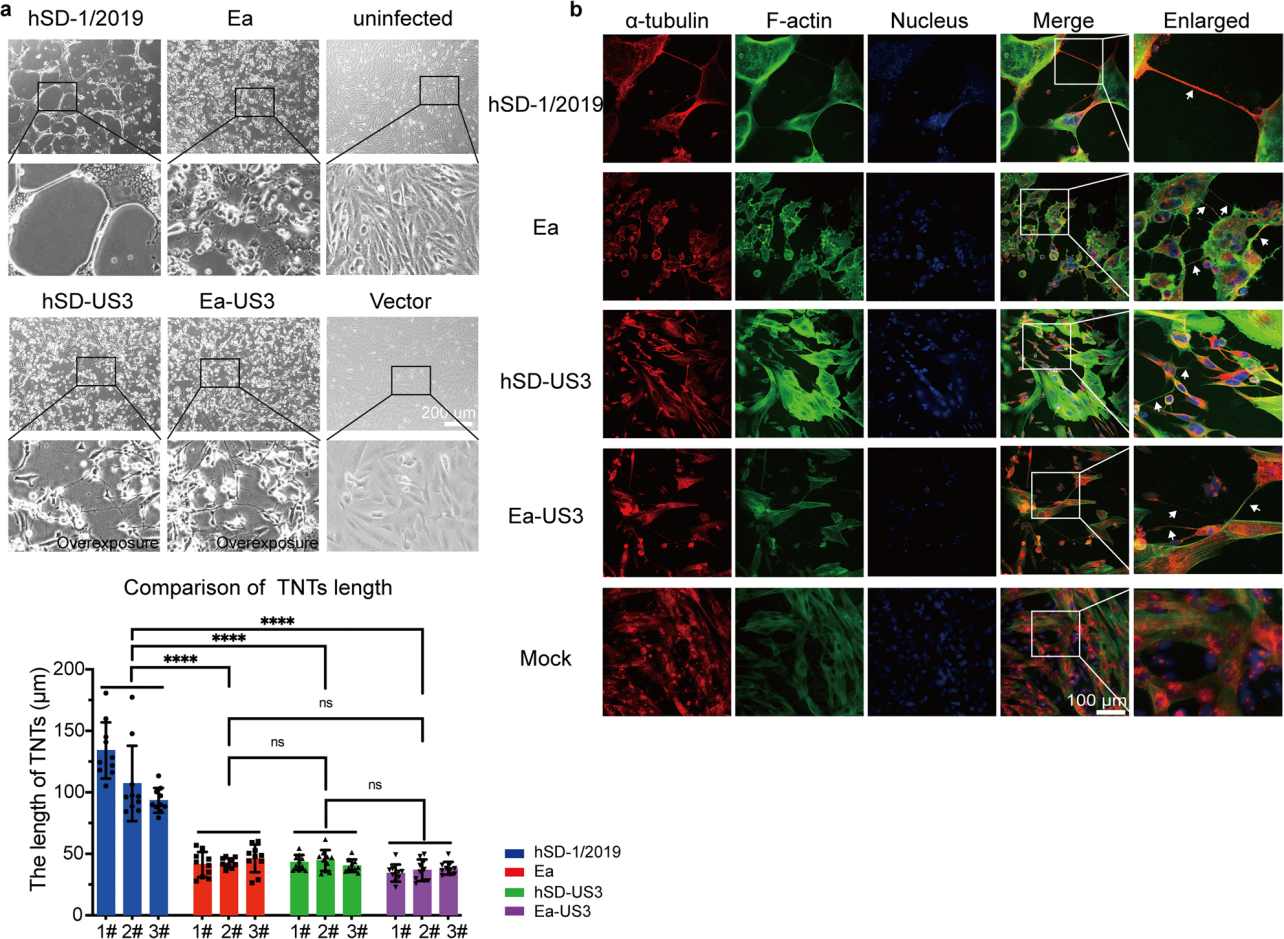
TNTs induced by US3 overexpression versus virus infection exhibit morphological differences. **a**. Brightfield images of TNTs induced by viral US3 overexpression versus PRV infection. The region in the smaller box was magnified in the images in the line below. The length of TNTs in cells infected with hSD-1/2019, Ea, hSDΔUS3 and EaΔUS3 strains were manually counted respectively in three separate experiments with 10 viewing fields randomly selected in each experiment. **b**. Immunofluorescence images of TNTs induced by viral US3 overexpression versus PRV infection. Immunofluorescence images were captured using a Nikon N-STORM microscope. US3: red; F-actin: green; nuclei: blue. Arrows indicate TNTs

Given that US3 is the essential driver of TNTogenesis, we hypothesized that the superior TNTs network induced by hSD-1/2019 might stem from functional differences in its US3 protein. To investigate this, we first compared the US3 protein sequences from both strains. Surprisingly, the amino acid sequences of the hSD-US3 and Ea-US3 proteins are 100% identical (Fig S4). Consistent with this genetic identity, transient overexpression of either hSD-US3 or Ea-US3 in SK-N-SH cells induced TNTs that were morphologically indistinguishable from each other in length and appearance (Fig. 7a). TNTs length was similar in Ea infection and US3 overexpression groups, which were significantly shorter than that in hSD-1/2019 infection group (Fig. 7a).

Crucially, the TNTs formed by US3 overexpression alone were uniformly short and curved, failing to recapitulate the long, straight, and thick phenotype characteristic of a full hSD-1/2019 infection (Fig. 7b). Taken together, these data reveal a two-part mechanism: while US3 is the essential initiator of TNTs formation, it is not solely responsible for the final morphology. This strongly suggests that other viral proteins, which differ between the variant and classical strains, act in concert with US3 to modulate and enhance the structure of the TNTs network, ultimately contributing to the hypervirulent phenotype of hSD-1/2019.

## Discussion

Lethal neuronal damage is a key event in PRV infection and pathogenicity. The mechanisms driving PRV’s potent neurotropism and neuropathogenicity, particularly in a non-natural host like humans, remain a critical knowledge gap. Considering that all known infected individuals exhibited neurological symptoms, this study utilized the PRV strain hSD-1/2019, previously isolated from a human case, to investigate the characteristics of PRV infection in neuronal cells. The objective was to elucidate the pathogenesis of PRV infection from the perspective of viral transmission. We demonstrate for the first time that PRV hijacks host cellular machinery to induce the formation of TNTs in SK-N-SH, SH-SY5Y, and mouse primary neurons. TNT structures act as viral superhighways, providing a novel mechanism for the rapid, long-distance dissemination of PRV, which might be important for the neuropathogenesis. Understanding the mechanisms underlying PRV transmission between neurons may provide potential therapeutic targets and contribute to the empirical and theoretical foundation for the effective treatment of human pseudorabies encephalitis.

During the viral replication cycle, mature PRV virions are released via budding and subsequently invade uninfected cells. It is also plausible that virions are directly transmitted through intercellular structures, such as TNTs, or via membrane fusion between adjacent cells. Previous studies have reported that PRV induces the formation of TNTs containing F-actin and tubulin in animal epithelial cells and fibroblasts (including ST, RK13, and MEF cells) [45, 47, 48]. In this study, we demonstrate that PRV is also capable of inducing TNTs formation in SK-N-SH cells. F-actin and α-tubulin were both detected in thick and elongated TNTs, whereas only F-actin was observed in thinner and shorter branches (Fig. 1e). These findings align with previous reports implying that stable microtubules might contribute to TNTs stability [47, 55]. In our time-lapse experiments, thick TNTs containing tubulin remained stable over a relatively long duration (12–24 h) (Video not shown). Furthermore, it has been reported that posttranslational modifications of the microtubules contributed to the stability of TNTs [47]. Here, infection of SK-N-SH cells with the hSD-1/2019 strain was found to induce longer and thicker TNTs compared to infection with the Ea strain (Fig. 6). Therefore, future studies should also compare the extent of posttranslational modifications in cells infected with these two PRV strains.

TNTs and other forms of intercellular junctions have been demonstrated to play a significant role in the transmission of various viruses, including HIV-1, murine leukemia virus, influenza virus, and SARS-CoV-2 [42, 44, 56–58]. In cells infected with hSD-1/2019 or hSD-mCherry where mCherry was fused to viral capsid protein VP26, fluorescent structures (either virions or capsids) were observed to move within elongations, and by in situ TEM, virions were observed being transferred between cells via TNTs (Fig. 4). Moreover, virus transmission from the infected donor cells to the uninfected receptor cells was not observed in the absence of TNTs induction (Fig. 5b). It has been proposed that during axonal transport, unenveloped capsids and glycoproteins—often encapsulated within vesicular structures—are transported independently [59]. Studies have shown that bovine herpesvirus 1 and herpes simplex virus type 1 can deliver viral proteins and genomic material directly into recipient cells through TNTs, leading to productive viral replication in previously uninfected cells [54, 60]. Additionally, intact virions have been detected within TNTs formed in ST cells following PRV infection [47]. Our findings suggest that TNTs contribute to both direct cell-to-cell and long-distance transmission of PRV in SK-N-SH cells.TEM analysis shows complete virions, as well as inclusion bodies, in the lumen of TNT (Fig. 4d). The specific viral components being transported via TNTs in SK-N-SH cells require further investigation in future studies.

Another key function of these TNTs is to facilitate immune evasion. Our co-culture experiments and in situ section TEM, conducted under immune pressure from neutralizing antibody with a complete neutralizing effect, reveal that TNTs serve as protected conduits for viral transmission. This mode of cell-to-cell spread allows PRV to bypass the host’s humoral immunity—specifically neutralizing antibodies—which are primary components of the defense against free virions. This protected pathway likely contributes to the establishment of persistent infection and may explain the virus’s ability to spread relentlessly within the central nervous system, even in the presence of an active immune response. This strategy is reminiscent of other successful pathogens like HIV, where TNTs-mediated transfer is in orders of magnitude more efficient than cell-free infection [57, 61]. It also draws parallels with recent findings for SARS-CoV-2, where TNTs were shown to mediate viral spread to neuronal cells lacking the canonical ACE2 receptor, offering a potential explanation for the neurological complications of COVID-19 [44].

Our findings reveal a sophisticated, two-part viral mechanism driving TNTs formation. We confirmed that the conserved alphaherpesvirus kinase US3 is the essential initiator of TNTogenesis in SK-N-SH cells. However, the observation that US3 overexpression alone fails to recapitulate the robust, mature TNTs phenotype seen during a full infection is highly significant. This discrepancy, coupled with the fact that the hypervirulent hSD-1/2019 strain produces a far superior TNTs network than the classical Ea strain despite possessing an identical US3 protein sequence, strongly argues that other viral factors are at play. These additional proteins, likely differing between the variant and classical strains, must act as crucial modulators, working in concert with US3 to regulate the length, thickness, and stability of the TNTs. This multi-protein strategy for shaping intercellular conduits is not unique to PRV. For instance, gammaherpesviruses, which lack a US3 homolog, utilize a complex of proteins to induce TNT-like protrusion structures, such as the gp48–ORF58 complex of murine gammaherpesvirus 68 (MHV-68) [62]. HIV has also been shown to induce TNTs through the activities of different viral proteins in different cell lines [57]. Here, our observations also indicate that there may be other viral proteins (in addition to US3) involved in the regulation of TNTs formation during PRV infection. Future studies should therefore focus on identifying these synergistic factors in PRV, which may include other viral proteins known to interact with the host cytoskeleton.

In addition to viral proteins, host factors also play a significant role in the formation of TNTs. In infected epithelial cells, the PRV US3 protein has been shown to activate the intracellular Cdc42/Rac1/PAK1 signaling pathway while simultaneously suppressing RhoA signaling, thereby promoting TNTs formation [45, 63]. Several studies have demonstrated that the HIV Nef protein facilitates TNTs formation in macrophages through interactions with PKA2, the exosome complex, and myosin-X (MyoX) [64–66]. MyoX has been identified as a positive regulator of TNTs formation in neurons [67] and is also identified in our study as one of the host proteins capable of interacting with US3 (data not shown). Therefore, to further elucidate the underlying mechanism of TNTs formation in SK-N-SH cells, additional studies are needed to identify the involved signal transduction networks, viral proteins (beyond US3), and host proteins (e.g., MyoX). Unraveling this complex signaling network is a key area of interest, as understanding how different viruses (e.g., HIV, Influenza, SARS-CoV-2) converge on or diverge from common host pathways to build these conduits holds immense value for developing broad-spectrum antiviral strategies.

## Conclusions

This study provides an association between intercellular structures and viral transmission, which might help explain the enhanced virulence of emerging human-infecting PRV variants. The ability of the hSD-1/2019 strain to engineer a denser, longer, and thicker TNTs network provides a compelling physical basis for its superior transmission efficiency. The differences in TNT morphology between the variant strain and classical strain exhibit a consistent trend with the differences in their viral transmission efficiency; further research is required to determine whether such differences are associated with neurovirulence and immune evasion capabilities. Viral US3 protein and its kinase activity are essential for TNT formation, and other unknown viral proteins are also involved in the maintenance of TNT morphology. Inhibiting the formation or function of TNT links could represent a host-directed therapeutic strategy, potentially effective not only against PRV but also against other neuroinvasive viruses that utilize similar mechanisms for pathogenesis.

## Supplementary Information

Supplementary Material 1: S1 Fig. Confirmation of PRV-US3 eukaryotic expression plasmids and kinase-inactivated US3 expression plasmids. a. Plasmid identification by PCR. The amplification products were of the expected size for the fragment of interest (1164 bp). b. Plasmid identification by double enzyme digestion. After double enzyme digestion, Ea-US3 and hSD-US3 plasmids showed vector fragment of 6249 bp and the target fragment of 1164 bp. c. Plasmid identification by sequencing. The fragment sequences of the US3 gene inserted in the plasmid were 100% homologous to those of the template. d. Identification of viral US3 expression by Western blot. Both Ea-US3 and hSD-US3 plasmids could express US3 protein in correct size. e. Kinase-inactivated US3 expression plasmids identification by sequencing. The fragment sequences of the kinase-inactivated US3 gene have undergone the correct mutation at the predetermined nucleotide loci. Compared with AAG sequence at positions 406–408 of the US3 gene, the nucleotide sequence at the same positions of the US3-136 gene has been replaced with GGT as designed. Compared with GAC sequence at positions 661-663 of the US3 gene, the nucleotide sequence at the same positions of the US3-221 gene has been replaced with GCT as designed. f. Identification of kinase-inactivated US3 protein expression by Western blot. Both kinase-inactive US3-136 and US3-221 plasmids could express US3 protein in correct size, with the US3-136 plasmid exhibiting relatively low expression levels. S2 Fig. TNTs fomation in neuronal-differentiated SK-N-SH cells following PRV infection. a. The differentiated SK-N-SH cells exhibited neuronal morphological characteristics, with small, round cell bodies and long axons. b. and c. The differentiated SK-N-SH cells expressed the neuronal markers TUBB3 (b) and MAP2 (c). d. hSD-1/2019 infection induced actin-rich filamentous structures that connect the dfferentiated neurons. Arrows indicate TNTs. S3 Fig. PRV hSD-1/2019 infection induced TNTs in SH-SY5Y and mouse primary neurons. a and b. hSD-1/2019 induced TNTs in SH-SY5Y cells. Arrows indicated TNTs. c and d. hSD-mCherry induced TNTs in mouse primary neurons. Arrows indicate TNTs. S4 Fig. Comparison of PRV US3 amino acid sequences between Ea and hSD-1/2019 strains.

Supplementary Material 2: S1 Video. Formation of filamentous structures in SK-N-SH cells infected with the PRV hSD-1/2019 strain. SK-N-SH cells were infected with the hSD-1/2019 strain at an MOI of 0.1. After 12 h, the cells were analyzed using an inverted fluorescence microscope (EVOS FL Auto). The movie consists of 120 frames captured over 10 h (1 frame/5 min) and shows the formation of filaments in SK-N-SH cells. S2 Video. The long filaments in SK-N-SH cells infected with the PRV hSD-1/2019 strain maintain stable existence. SK-N-SH cells were infected with the hSD-1/2019 strain at an MOI of 0.1. After 24 h, the cells were analyzed using an inverted fluorescence microscope (EVOS FL Auto). The movie consists of 11 frames captured over 150 min (1 frame/15 min) and shows the formation of filaments in SK-N-SH cells. S3 Video. Formation of filamentous structures in SK-N-SH cells transfected with PRV-US3 plasmids. SK-N-SH cells were transfected with the Ea-US3 plasmid. After 24 h, the cells were analyzed using an inverted fluorescence microscope (EVOS FL Auto). The movie consists of 30 frames captured over 5 h (1 frame/10 min) and shows the formation of filaments in SK-N-SH cells. S4 and S5 Videos. Transport of EGFP through TNTs induced by hSD-US3 (S3 Video) and Ea-US3 (S4 Video) plasmids transfection. Plasmids containing viral US3 and EGFP were co-transfected in SK-N-SH cells, respectively. After 24 h, the cells were analyzed using an inverted fluorescence microscope (EVOS FL Auto). The movie in S4 video consists of 30 frames captured over 5 h (1 frame/10 min) and the movie in S5 video consists of 61 frames captured over 10 h (1 frame/10 min), showing that US3-induced TNTs mediate the transport of EGFP between cells. S6 Video. Transport of virions through the tubular structures of TNTs. SK-N-SH cells were infected with hSD-mCherry virions at an MOI of 0.1. After 24 h, the cells were analyzed using a confocal microscope (ZEISS LSM 800). The movie consists of 40 frames captured over 200 min (1 frame/5 min) and shows mCherry-labeled virions being transmitted between cells through the tubular structures of TNTs. S7 Video. Intercellular transport of multiple vesicular structures within TNTs. SK-N-SH cells were infected with hSD-mCherry virions at an MOI of 0.1. After 24 h, the cells were analyzed using an inverted fluorescence microscope (EVOS FL Auto). The movie consists of 68 frames captured over 340 min (1 frame/5 min) and shows multiple vesicular structures being rapidly transported between cells within TNTs. S8 Video. Transport of virions through vesicular structures within TNTs. SK-N-SH cells were infected with hSD-mCherry virions at an MOI of 0.1. After 24 h, the cells were analyzed using a confocal microscope (ZEISS LSM 800). The movie consists of 25 frames captured over 120 min (1 frame/5 min) and shows mCherry-labeled virions being transmitted via the movement of vesicular structures within TNTs.

## Data Availability

All data generated or analyzed during this study are included in this article and its supplementary information files. Further datasets are available from the corresponding author upon reasonable request. Source data for figures and analyses are provided with this paper.

## Abbreviations

PRV: Pseudorabies virus
TNTs: Tunneling nanotubes
US3: The conserved alphaherpesvirus kinase
hSD-1/2019: A human-isolate variant PRV strain
Ea: A pig-isolate classical PRV strain
HIV: Human immunodeficiency virus
SARS-CoV-2: Severe acute respiratory syndrome coronavirus 2
SK-N-SH: Human neuroblastoma cells
PK-15: Porcine kidney cells
FBS: Fetal bovine serum
mCherry: Monomeric Cherry red fluorescent protein
EGFP: Enhanced Green Fluorescent Protein
hSD-mCherry and Ea-mCherry: The recombinant PRV-mCherry strains
hSDΔUS3 and EaΔUS3: The US3-knockout PRV strains
MOI: Multiplicity of infection
PBS: Phosphate-buffered saline
gB: Viral glycoprotein B
VP26: Viral capsid protein 26
ORF: Open reading frame
PCR: Polymerase Chain Reaction
hSD-US3 and Ea-US3: Expression of US3 plasmids
MyoX: Myosin-X
