# Supplementary figures and images for: Tunneling nanotubes induced by pseudorabies virus facilitate viral transmission in neuronal cells to evade the immune system
Source: Cell Commun Signal. 2026 Feb 28;24:208. doi: 10.1186/s12964-026-02765-8 (PMC13059447; doi:10.1186/s12964-026-02765-8)

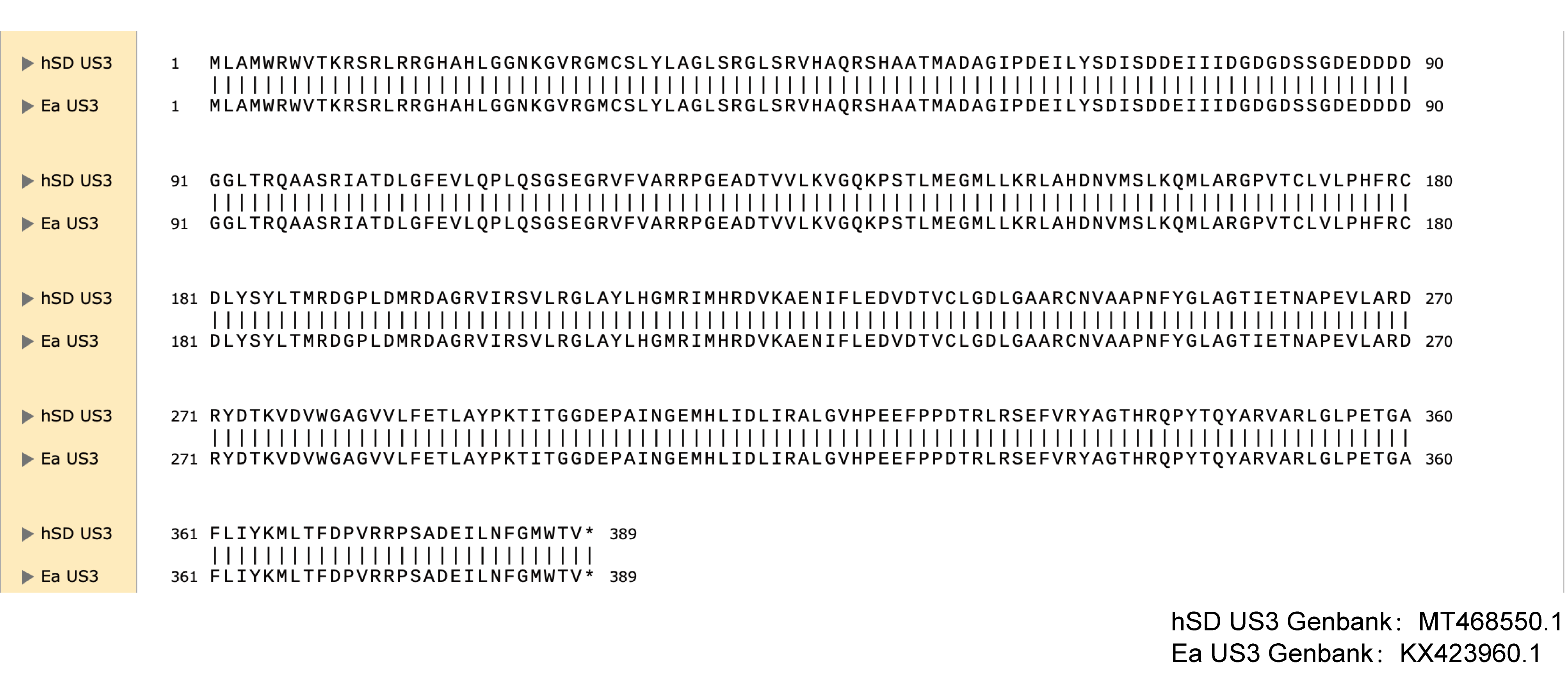

Supplement: Supplementary file 1 — Supplementary Material 1: S1 Fig. Confirmation of PRV-US3 eukaryotic expression plasmids and kinase-inactivated US3 expression plasmids. a. Plasmid identification by PCR. The amplification products were of the expected size for the fragment of interest (1164 bp). b. Plasmid identification by double enzyme digestion. After double enzyme digestion, Ea-US3 and hSD-US3 plasmids showed vector fragment of 6249 bp and the target fragment of 1164 bp. c. Plasmid identification by sequencing. The fragment sequences of the US3 gene inserted in the plasmid were 100% homologous to those of the template. d. Identification of viral US3 expression by Western blot. Both Ea-US3 and hSD-US3 plasmids could express US3 protein in correct size. e. Kinase-inactivated US3 expression plasmids identification by sequencing. The fragment sequences of the kinase-inactivated US3 gene have undergone the correct mutation at the predetermined nucleotide loci. Compared with AAG sequence at positions 406–408 of the US3 gene, the nucleotide sequence at the same positions of the US3-136 gene has been replaced with GGT as designed. Compared with GAC sequence at positions 661-663 of the US3 gene, the nucleotide sequence at the same positions of the US3-221 gene has been replaced with GCT as designed. f. Identification of kinase-inactivated US3 protein expression by Western blot. Both kinase-inactive US3-136 and US3-221 plasmids could express US3 protein in correct size, with the US3-136 plasmid exhibiting relatively low expression levels. S2 Fig. TNTs fomation in neuronal-differentiated SK-N-SH cells following PRV infection. a. The differentiated SK-N-SH cells exhibited neuronal morphological characteristics, with small, round cell bodies and long axons. b. and c. The differentiated SK-N-SH cells expressed the neuronal markers TUBB3 (b) and MAP2 (c). d. hSD-1/2019 infection induced actin-rich filamentous structures that connect the dfferentiated neurons. Arrows indicate TNTs. S3 Fig. PRV hSD-1/2019 infec [file 12964_2026_2765_MOESM1_ESM.zip › Revised Figure S4.tif]

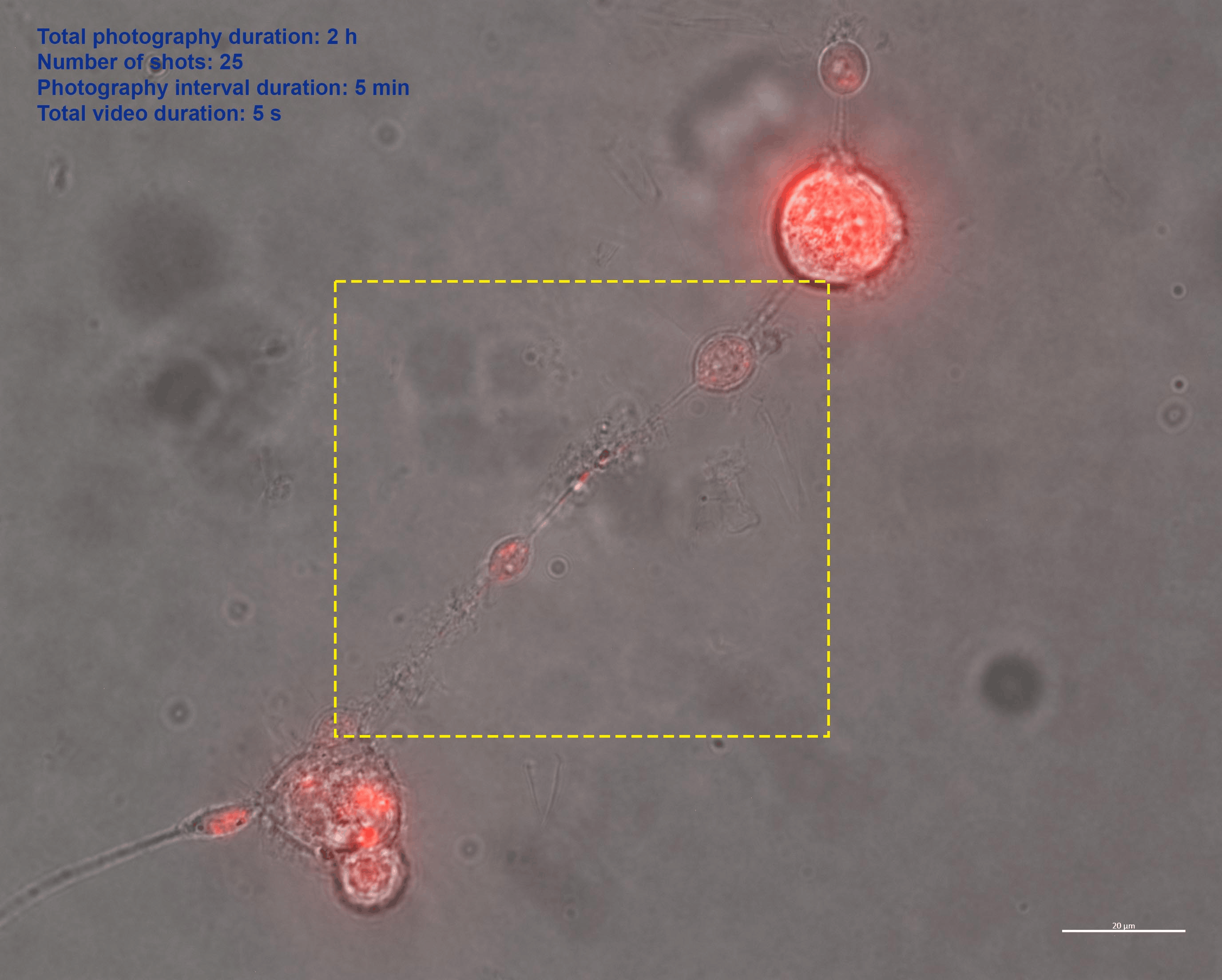

Supplement: Supplementary file 2 — Supplementary Material 2: S1 Video. Formation of filamentous structures in SK-N-SH cells infected with the PRV hSD-1/2019 strain. SK-N-SH cells were infected with the hSD-1/2019 strain at an MOI of 0.1. After 12 h, the cells were analyzed using an inverted fluorescence microscope (EVOS FL Auto). The movie consists of 120 frames captured over 10 h (1 frame/5 min) and shows the formation of filaments in SK-N-SH cells. S2 Video. The long filaments in SK-N-SH cells infected with the PRV hSD-1/2019 strain maintain stable existence. SK-N-SH cells were infected with the hSD-1/2019 strain at an MOI of 0.1. After 24 h, the cells were analyzed using an inverted fluorescence microscope (EVOS FL Auto). The movie consists of 11 frames captured over 150 min (1 frame/15 min) and shows the formation of filaments in SK-N-SH cells. S3 Video. Formation of filamentous structures in SK-N-SH cells transfected with PRV-US3 plasmids. SK-N-SH cells were transfected with the Ea-US3 plasmid. After 24 h, the cells were analyzed using an inverted fluorescence microscope (EVOS FL Auto). The movie consists of 30 frames captured over 5 h (1 frame/10 min) and shows the formation of filaments in SK-N-SH cells. S4 and S5 Videos. Transport of EGFP through TNTs induced by hSD-US3 (S3 Video) and Ea-US3 (S4 Video) plasmids transfection. Plasmids containing viral US3 and EGFP were co-transfected in SK-N-SH cells, respectively. After 24 h, the cells were analyzed using an inverted fluorescence microscope (EVOS FL Auto). The movie in S4 video consists of 30 frames captured over 5 h (1 frame/10 min) and the movie in S5 video consists of 61 frames captured over 10 h (1 frame/10 min), showing that US3-induced TNTs mediate the transport of EGFP between cells. S6 Video. Transport of virions through the tubular structures of TNTs. SK-N-SH cells were infected with hSD-mCherry virions at an MOI of 0.1. After 24 h, the cells were analyzed using a confocal microscope (ZEISS LSM 800). The movie consists of 40 frames c [file 12964_2026_2765_MOESM2_ESM.zip › S8 Video.gif]

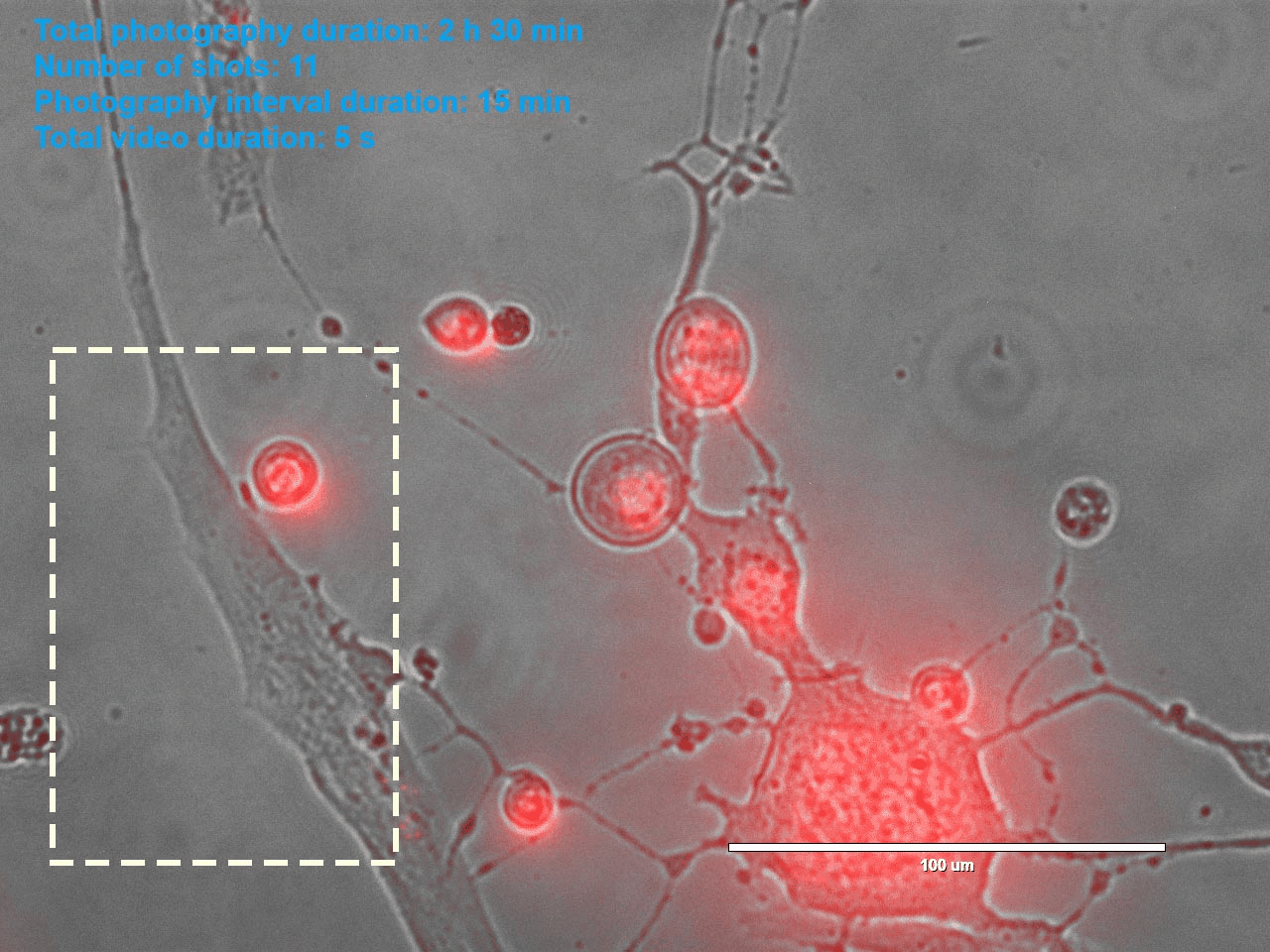

Supplement: Supplementary file 2 — Supplementary Material 2: S1 Video. Formation of filamentous structures in SK-N-SH cells infected with the PRV hSD-1/2019 strain. SK-N-SH cells were infected with the hSD-1/2019 strain at an MOI of 0.1. After 12 h, the cells were analyzed using an inverted fluorescence microscope (EVOS FL Auto). The movie consists of 120 frames captured over 10 h (1 frame/5 min) and shows the formation of filaments in SK-N-SH cells. S2 Video. The long filaments in SK-N-SH cells infected with the PRV hSD-1/2019 strain maintain stable existence. SK-N-SH cells were infected with the hSD-1/2019 strain at an MOI of 0.1. After 24 h, the cells were analyzed using an inverted fluorescence microscope (EVOS FL Auto). The movie consists of 11 frames captured over 150 min (1 frame/15 min) and shows the formation of filaments in SK-N-SH cells. S3 Video. Formation of filamentous structures in SK-N-SH cells transfected with PRV-US3 plasmids. SK-N-SH cells were transfected with the Ea-US3 plasmid. After 24 h, the cells were analyzed using an inverted fluorescence microscope (EVOS FL Auto). The movie consists of 30 frames captured over 5 h (1 frame/10 min) and shows the formation of filaments in SK-N-SH cells. S4 and S5 Videos. Transport of EGFP through TNTs induced by hSD-US3 (S3 Video) and Ea-US3 (S4 Video) plasmids transfection. Plasmids containing viral US3 and EGFP were co-transfected in SK-N-SH cells, respectively. After 24 h, the cells were analyzed using an inverted fluorescence microscope (EVOS FL Auto). The movie in S4 video consists of 30 frames captured over 5 h (1 frame/10 min) and the movie in S5 video consists of 61 frames captured over 10 h (1 frame/10 min), showing that US3-induced TNTs mediate the transport of EGFP between cells. S6 Video. Transport of virions through the tubular structures of TNTs. SK-N-SH cells were infected with hSD-mCherry virions at an MOI of 0.1. After 24 h, the cells were analyzed using a confocal microscope (ZEISS LSM 800). The movie consists of 40 frames c [file 12964_2026_2765_MOESM2_ESM.zip › S2 Video.gif]

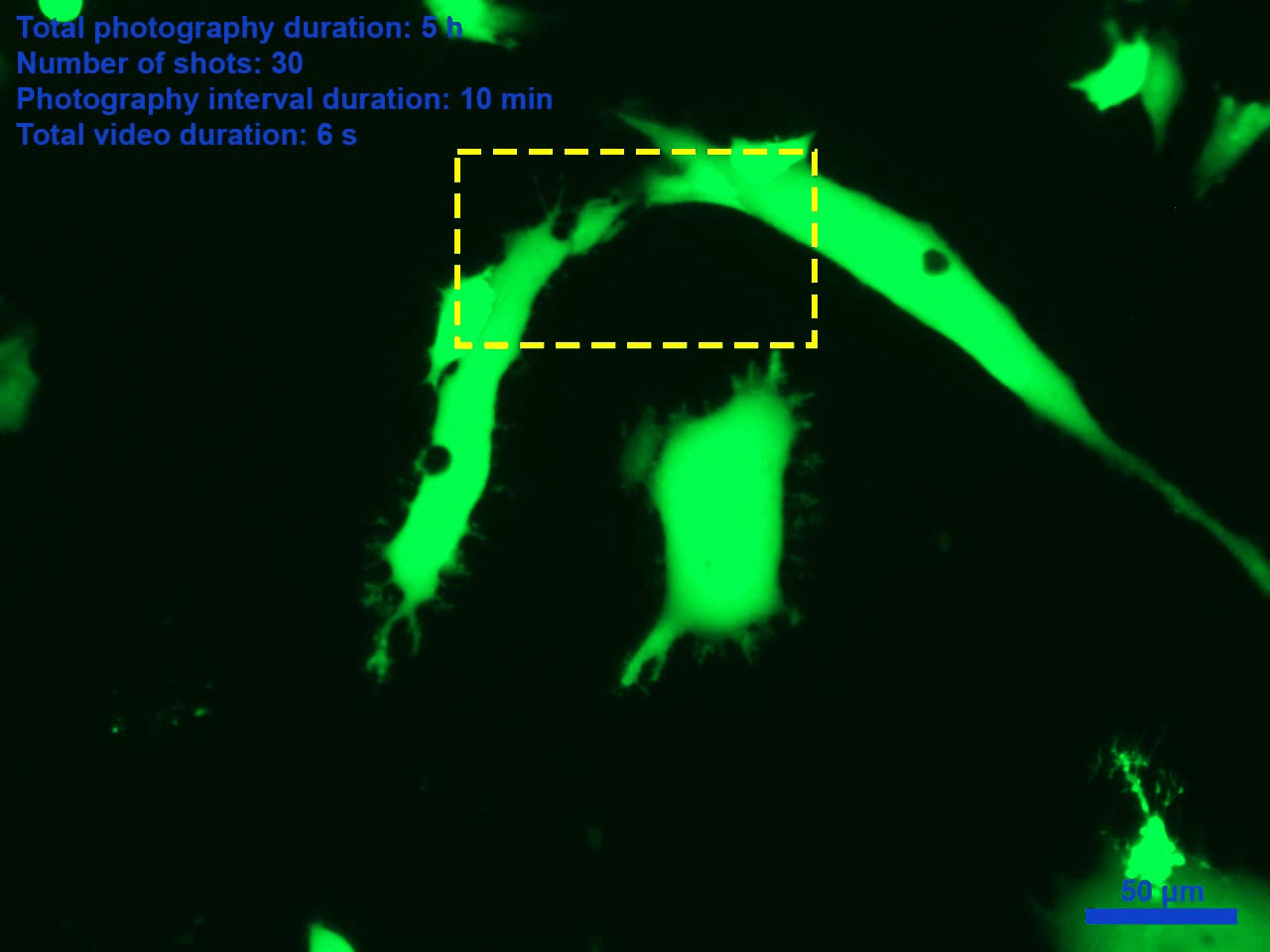

Supplement: Supplementary file 2 — Supplementary Material 2: S1 Video. Formation of filamentous structures in SK-N-SH cells infected with the PRV hSD-1/2019 strain. SK-N-SH cells were infected with the hSD-1/2019 strain at an MOI of 0.1. After 12 h, the cells were analyzed using an inverted fluorescence microscope (EVOS FL Auto). The movie consists of 120 frames captured over 10 h (1 frame/5 min) and shows the formation of filaments in SK-N-SH cells. S2 Video. The long filaments in SK-N-SH cells infected with the PRV hSD-1/2019 strain maintain stable existence. SK-N-SH cells were infected with the hSD-1/2019 strain at an MOI of 0.1. After 24 h, the cells were analyzed using an inverted fluorescence microscope (EVOS FL Auto). The movie consists of 11 frames captured over 150 min (1 frame/15 min) and shows the formation of filaments in SK-N-SH cells. S3 Video. Formation of filamentous structures in SK-N-SH cells transfected with PRV-US3 plasmids. SK-N-SH cells were transfected with the Ea-US3 plasmid. After 24 h, the cells were analyzed using an inverted fluorescence microscope (EVOS FL Auto). The movie consists of 30 frames captured over 5 h (1 frame/10 min) and shows the formation of filaments in SK-N-SH cells. S4 and S5 Videos. Transport of EGFP through TNTs induced by hSD-US3 (S3 Video) and Ea-US3 (S4 Video) plasmids transfection. Plasmids containing viral US3 and EGFP were co-transfected in SK-N-SH cells, respectively. After 24 h, the cells were analyzed using an inverted fluorescence microscope (EVOS FL Auto). The movie in S4 video consists of 30 frames captured over 5 h (1 frame/10 min) and the movie in S5 video consists of 61 frames captured over 10 h (1 frame/10 min), showing that US3-induced TNTs mediate the transport of EGFP between cells. S6 Video. Transport of virions through the tubular structures of TNTs. SK-N-SH cells were infected with hSD-mCherry virions at an MOI of 0.1. After 24 h, the cells were analyzed using a confocal microscope (ZEISS LSM 800). The movie consists of 40 frames c [file 12964_2026_2765_MOESM2_ESM.zip › S3 Video.gif]

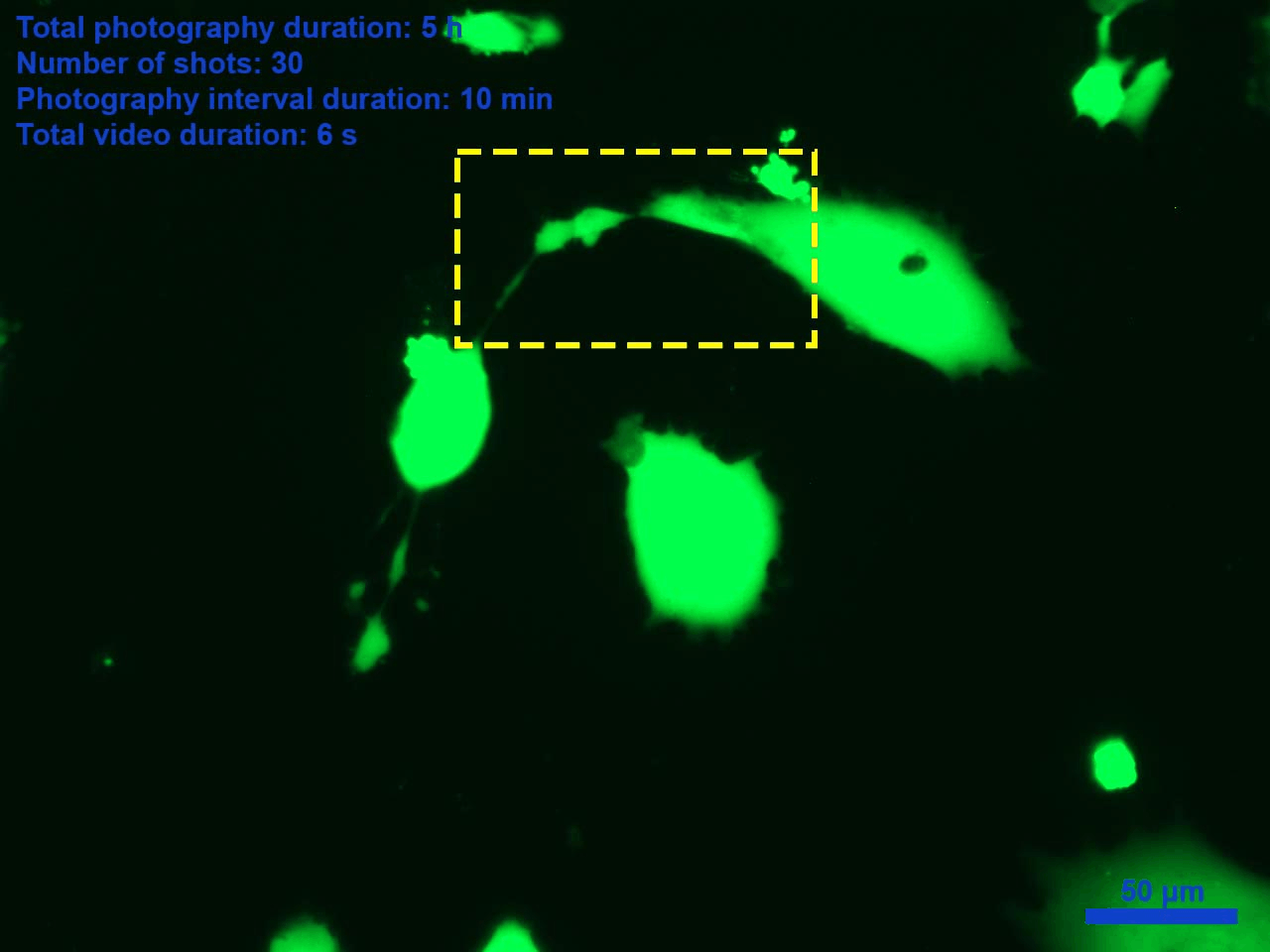

Supplement: Supplementary file 2 — Supplementary Material 2: S1 Video. Formation of filamentous structures in SK-N-SH cells infected with the PRV hSD-1/2019 strain. SK-N-SH cells were infected with the hSD-1/2019 strain at an MOI of 0.1. After 12 h, the cells were analyzed using an inverted fluorescence microscope (EVOS FL Auto). The movie consists of 120 frames captured over 10 h (1 frame/5 min) and shows the formation of filaments in SK-N-SH cells. S2 Video. The long filaments in SK-N-SH cells infected with the PRV hSD-1/2019 strain maintain stable existence. SK-N-SH cells were infected with the hSD-1/2019 strain at an MOI of 0.1. After 24 h, the cells were analyzed using an inverted fluorescence microscope (EVOS FL Auto). The movie consists of 11 frames captured over 150 min (1 frame/15 min) and shows the formation of filaments in SK-N-SH cells. S3 Video. Formation of filamentous structures in SK-N-SH cells transfected with PRV-US3 plasmids. SK-N-SH cells were transfected with the Ea-US3 plasmid. After 24 h, the cells were analyzed using an inverted fluorescence microscope (EVOS FL Auto). The movie consists of 30 frames captured over 5 h (1 frame/10 min) and shows the formation of filaments in SK-N-SH cells. S4 and S5 Videos. Transport of EGFP through TNTs induced by hSD-US3 (S3 Video) and Ea-US3 (S4 Video) plasmids transfection. Plasmids containing viral US3 and EGFP were co-transfected in SK-N-SH cells, respectively. After 24 h, the cells were analyzed using an inverted fluorescence microscope (EVOS FL Auto). The movie in S4 video consists of 30 frames captured over 5 h (1 frame/10 min) and the movie in S5 video consists of 61 frames captured over 10 h (1 frame/10 min), showing that US3-induced TNTs mediate the transport of EGFP between cells. S6 Video. Transport of virions through the tubular structures of TNTs. SK-N-SH cells were infected with hSD-mCherry virions at an MOI of 0.1. After 24 h, the cells were analyzed using a confocal microscope (ZEISS LSM 800). The movie consists of 40 frames c [file 12964_2026_2765_MOESM2_ESM.zip › S4 Video.gif]

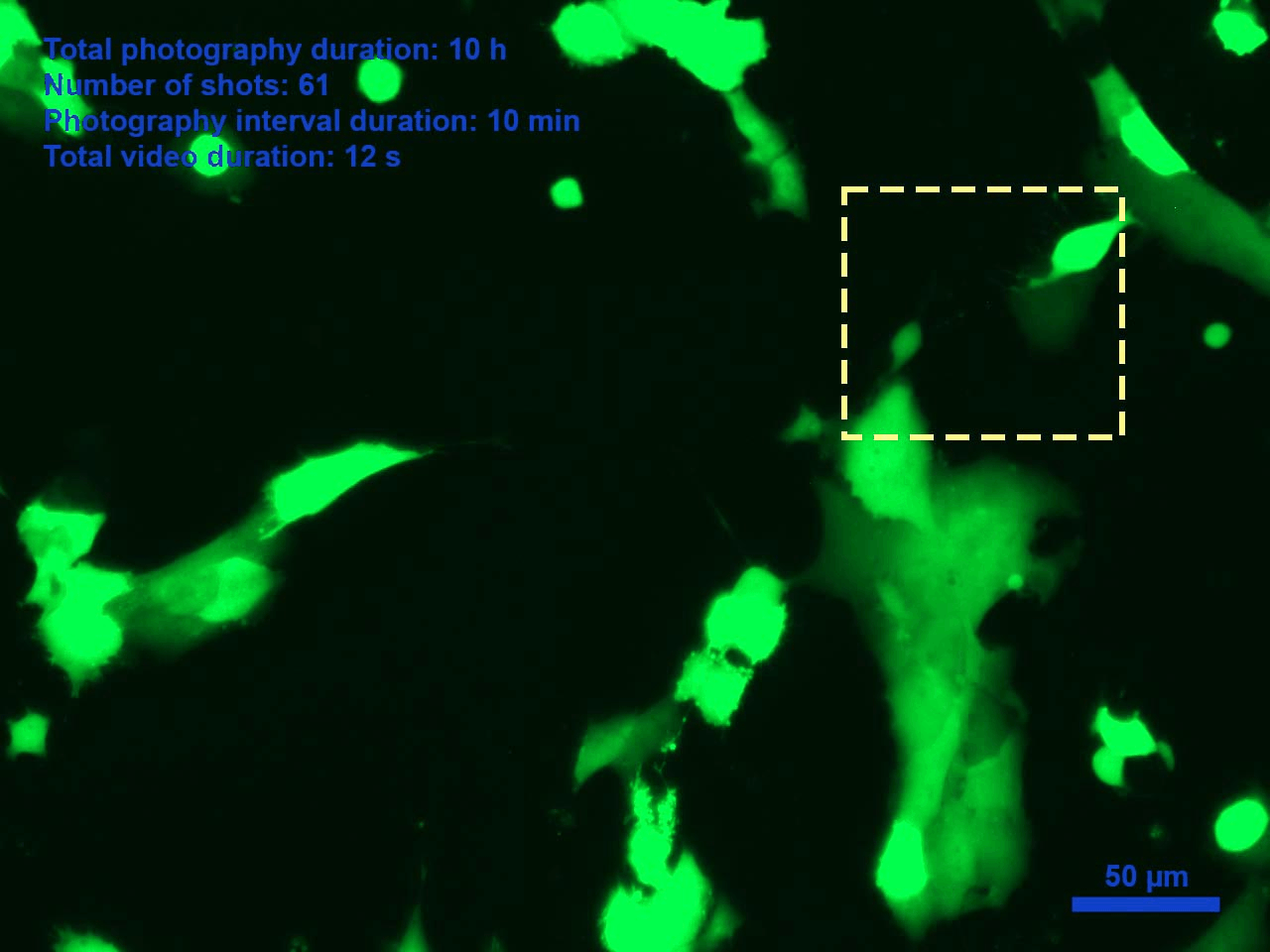

Supplement: Supplementary file 2 — Supplementary Material 2: S1 Video. Formation of filamentous structures in SK-N-SH cells infected with the PRV hSD-1/2019 strain. SK-N-SH cells were infected with the hSD-1/2019 strain at an MOI of 0.1. After 12 h, the cells were analyzed using an inverted fluorescence microscope (EVOS FL Auto). The movie consists of 120 frames captured over 10 h (1 frame/5 min) and shows the formation of filaments in SK-N-SH cells. S2 Video. The long filaments in SK-N-SH cells infected with the PRV hSD-1/2019 strain maintain stable existence. SK-N-SH cells were infected with the hSD-1/2019 strain at an MOI of 0.1. After 24 h, the cells were analyzed using an inverted fluorescence microscope (EVOS FL Auto). The movie consists of 11 frames captured over 150 min (1 frame/15 min) and shows the formation of filaments in SK-N-SH cells. S3 Video. Formation of filamentous structures in SK-N-SH cells transfected with PRV-US3 plasmids. SK-N-SH cells were transfected with the Ea-US3 plasmid. After 24 h, the cells were analyzed using an inverted fluorescence microscope (EVOS FL Auto). The movie consists of 30 frames captured over 5 h (1 frame/10 min) and shows the formation of filaments in SK-N-SH cells. S4 and S5 Videos. Transport of EGFP through TNTs induced by hSD-US3 (S3 Video) and Ea-US3 (S4 Video) plasmids transfection. Plasmids containing viral US3 and EGFP were co-transfected in SK-N-SH cells, respectively. After 24 h, the cells were analyzed using an inverted fluorescence microscope (EVOS FL Auto). The movie in S4 video consists of 30 frames captured over 5 h (1 frame/10 min) and the movie in S5 video consists of 61 frames captured over 10 h (1 frame/10 min), showing that US3-induced TNTs mediate the transport of EGFP between cells. S6 Video. Transport of virions through the tubular structures of TNTs. SK-N-SH cells were infected with hSD-mCherry virions at an MOI of 0.1. After 24 h, the cells were analyzed using a confocal microscope (ZEISS LSM 800). The movie consists of 40 frames c [file 12964_2026_2765_MOESM2_ESM.zip › S5 Video.gif]

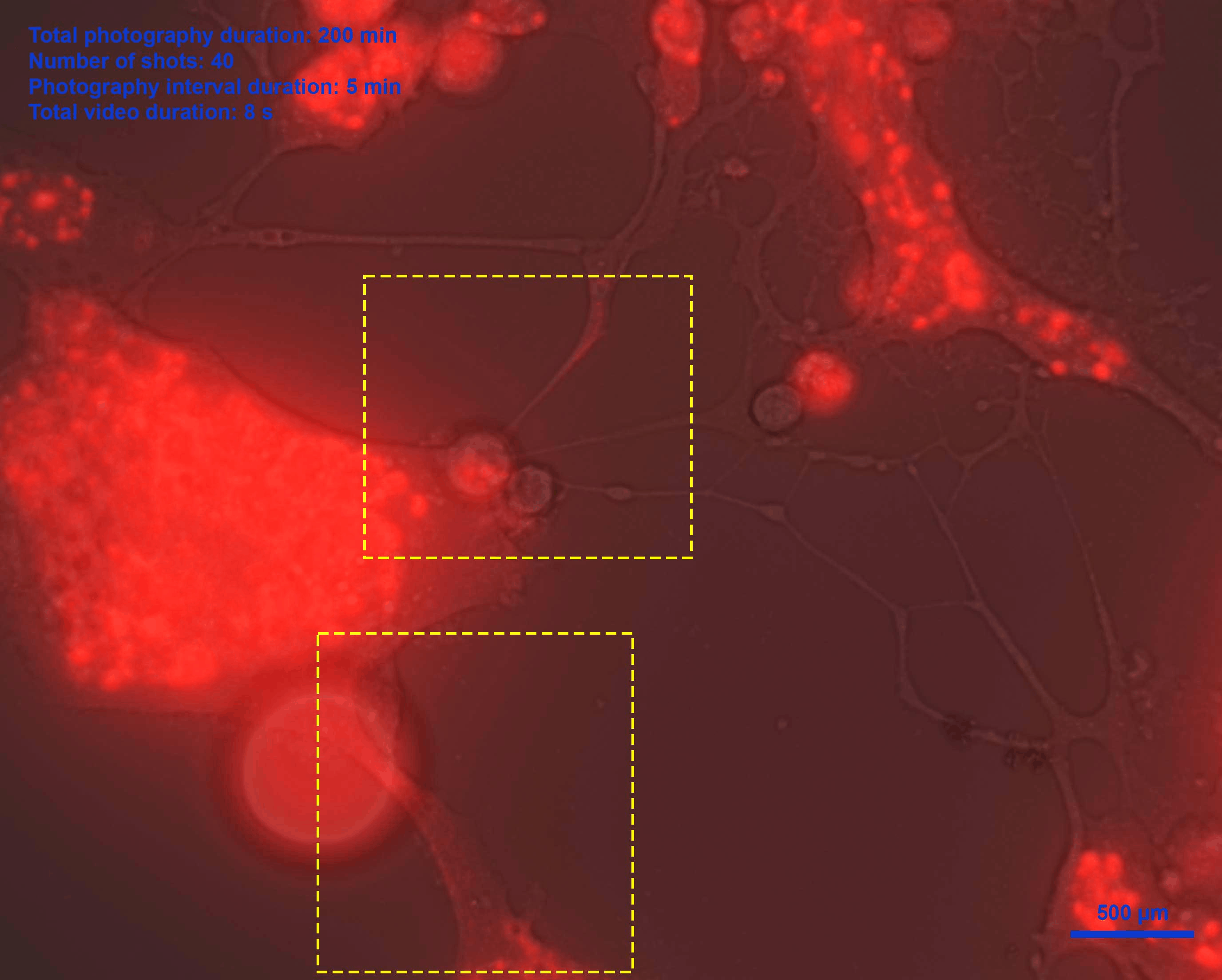

Supplement: Supplementary file 2 — Supplementary Material 2: S1 Video. Formation of filamentous structures in SK-N-SH cells infected with the PRV hSD-1/2019 strain. SK-N-SH cells were infected with the hSD-1/2019 strain at an MOI of 0.1. After 12 h, the cells were analyzed using an inverted fluorescence microscope (EVOS FL Auto). The movie consists of 120 frames captured over 10 h (1 frame/5 min) and shows the formation of filaments in SK-N-SH cells. S2 Video. The long filaments in SK-N-SH cells infected with the PRV hSD-1/2019 strain maintain stable existence. SK-N-SH cells were infected with the hSD-1/2019 strain at an MOI of 0.1. After 24 h, the cells were analyzed using an inverted fluorescence microscope (EVOS FL Auto). The movie consists of 11 frames captured over 150 min (1 frame/15 min) and shows the formation of filaments in SK-N-SH cells. S3 Video. Formation of filamentous structures in SK-N-SH cells transfected with PRV-US3 plasmids. SK-N-SH cells were transfected with the Ea-US3 plasmid. After 24 h, the cells were analyzed using an inverted fluorescence microscope (EVOS FL Auto). The movie consists of 30 frames captured over 5 h (1 frame/10 min) and shows the formation of filaments in SK-N-SH cells. S4 and S5 Videos. Transport of EGFP through TNTs induced by hSD-US3 (S3 Video) and Ea-US3 (S4 Video) plasmids transfection. Plasmids containing viral US3 and EGFP were co-transfected in SK-N-SH cells, respectively. After 24 h, the cells were analyzed using an inverted fluorescence microscope (EVOS FL Auto). The movie in S4 video consists of 30 frames captured over 5 h (1 frame/10 min) and the movie in S5 video consists of 61 frames captured over 10 h (1 frame/10 min), showing that US3-induced TNTs mediate the transport of EGFP between cells. S6 Video. Transport of virions through the tubular structures of TNTs. SK-N-SH cells were infected with hSD-mCherry virions at an MOI of 0.1. After 24 h, the cells were analyzed using a confocal microscope (ZEISS LSM 800). The movie consists of 40 frames c [file 12964_2026_2765_MOESM2_ESM.zip › S6 Video.gif]
